# Unveiling the mechanistic link between extracellular amyloid fibrils, mechano-signaling and YAP activation in cancer

**DOI:** 10.1038/s41419-024-06424-z

**Published:** 2024-01-11

**Authors:** Francesco Farris, Alice Elhagh, Ilaria Vigorito, Nicoletta Alongi, Federica Pisati, Michele Giannattasio, Francesca Casagrande, Lisa Veghini, Vincenzo Corbo, Claudio Tripodo, Arianna Di Napoli, Vittoria Matafora, Angela Bachi

**Affiliations:** 1 IFOM ETS – The AIRC Institute of Molecular Oncology, 20139 Milan, Italy; 2 Histopathology Unit, Cogentech S.C.a.R.L, 20139 Milan, Italy; 3 Department of Oncology and Hemato-Oncology, University of Milan, 20122 Milan, Italy; 4 Department of Engineering for Innovation Medicine (DIMI), University of Verona, 37134 Verona, Italy; 5 ARC-Net Centre for Applied Research on Cancer, University of Verona, 37134 Verona, Italy; 6 Tumor Immunology Unit, Department of Health Sciences, University of Palermo, 90133 Palermo, Italy; 7 Pathology Unit, Department of Clinical and Molecular Medicine, Sant’Andrea University Hospital, Sapienza University of Rome, 00189 Rome, Italy; 8 Present Address: IRCCS Humanitas Research Hospital, Rozzano, Milan, Italy; 9 Present Address: Human Technopole, Milan, Italy

**Keywords:** Cancer microenvironment, Proteomics, Cell signalling

## Abstract

The tumor microenvironment is a complex ecosystem that plays a critical role in cancer progression and treatment response. Recently, extracellular amyloid fibrils have emerged as novel components of the tumor microenvironment; however, their function remains elusive. In this study, we establish a direct connection between the presence of amyloid fibrils in the secretome and the activation of YAP, a transcriptional co-activator involved in cancer proliferation and drug resistance. Furthermore, we uncover a shared mechano-signaling mechanism triggered by amyloid fibrils in both melanoma and pancreatic ductal adenocarcinoma cells. Our findings highlight the crucial role of the glycocalyx protein Agrin which binds to extracellular amyloid fibrils and acts as a necessary factor in driving amyloid-dependent YAP activation. Additionally, we reveal the involvement of the HIPPO pathway core kinase LATS1 in this signaling cascade. Finally, we demonstrate that extracellular amyloid fibrils enhance cancer cell migration and invasion. In conclusion, our research expands our knowledge of the tumor microenvironment by uncovering the role of extracellular amyloid fibrils in driving mechano-signaling and YAP activation. This knowledge opens up new avenues for developing innovative strategies to modulate YAP activation and mitigate its detrimental effects during cancer progression.

## Introduction

In recent years, Tumor Microenvironment (TME) captured the attention of the scientific community due to its capacity to contribute to cancer progression by affecting proliferation, invasion, drug resistance, and immune evasion [1–3]. TME is composed of different types of cells such as stromal cells, immune cells, adipocytes, endothelial cells, and acellular components such as soluble factors and the extracellular matrix (ECM) [4]. The soluble factors play different roles inside the TME. Cancers-secreted soluble factors can have autocrine activity, meaning that they can affect surrounding cancer cells, or paracrine effects, recruiting different types of cells such as immune cells or fibroblasts with pro-tumorigenic activity [5]. By recruiting and activating TAMs and Tregs cells, cancer cells create a local immunosuppressive environment that helps them evade the immune system’s surveillance [6, 7], while cancer-associated fibroblasts (CAFs) promote the deposition of ECM, creating a safe-haven that protects cells from chemotherapy [8, 9]. Research on the cancer secretome and the associated signaling cascades has indeed emerged as pivotal in the quest for understanding, and subsequent inhibition of tumor progression. We analyzed the secretome of primitive and metastatic melanoma cells to understand the differences in the secretome of cancer cells at different stages of the disease [10]. In our previous work, we discovered that metastatic melanoma cells secrete different amyloidogenic proteins such as APP and PMEL, and proteins that assist amyloidogenesis such as APOE, SORT1, and QPCT [10]. We also verified amyloid accumulation in biopsies from metastatic melanoma patients. Mechanistically, we demonstrated that extracellular amyloid fibrils increase YAP activation in an autocrine fashion [10]. YAP (Yes Associated Protein) is known to play pivotal roles in cancer progression and it is activated by ECM stiffness and mechanical stimuli [11]. Cancer cells perceive ECM stiffness through integrins and focal adhesions, which exert forces against the ECM [12]. These forces cause actin cytoskeleton remodeling and/or HIPPO pathway deactivation, thus promoting YAP nuclear translocation together with its transcriptional co-activator TAZ [13]. YAP interacts with the TEAD family of transcription factors, controlling the expression of their target genes such as CTGF and CYR61 [14]. YAP and its target genes promote cancer initiation, progression and metastasis formation through the regulation of proliferation, migration, invasion, drug resistance, epithelial to mesenchymal transition (EMT), and stem cell maintenance [15]. Due to the detrimental effects of YAP transcriptional activity, several drugs that displace the interaction between YAP and TEAD, such as Verteporfin [16], Peptide17 [17] or CA3 [18], have been developed but their efficacy in clinical trials still has to be proven. It has been described that YAP can be activated by increased stiffness of the ECM or by remodeling of the actin cytoskeleton, but how cancer-secreted factors can influence YAP activity remains a poorly understood process. Therefore, a deeper understanding of the intricate molecular network that leads to YAP activation still represents an open question. We reasoned that cancer-secreted amyloid fibrils, thanks to their intrinsic rigidity [19], could mimic a mechanical stimulus, thus representing a molecular trigger for mechanosignalling. Coherently with our findings [10], previous studies have found that Amyloid β40 (Aβ40) and Amyloid β42 (Aβ42) peptides, the amyloid peptides deriving from APP, are enriched in breast cancer tissues [20] and in plasma of patients affected by cancer [21]. These studies correlated the presence of amyloid species with worse cancer progression without defining any functional link between the presence of amyloid and the pathogenesis or progression of the disease. In more recent years, some research groups tried to build a mechanistic link between the presence of amyloids and cancer progression. One of those studies regards the well-known tumor suppressor p53, which exhibits amyloidogenic tendencies in several tumors. Multiple factors contribute to the aggregation of p53, including mutations in specific domains predisposed to aggregation, the role of chaperone molecules, pH levels, and the availability of ions such as Zn^2+^ [22]. p53 aggregates determine both loss and gain of function effects; indeed, p53 loses its effects on cell cycle arrest and apoptosis and gain oncogenic effects increasing cell proliferation, cell migration and malignant transformation [22]. In a distinct work conducted by Munir et al., with a particular focus on extracellular amyloid aggregation, it was demonstrated that Amyloid β accumulates within the stroma of melanoma and pancreatic cancer patients. This accumulation, in turn, facilitates the release of Neutrophil Extracellular Traps (NETs), stimulating tumor growth and metastasis formation [23]. Further, it was demonstrated that, in a melanoma mouse model, melanoma-secreted Amyloid β suppresses neuroinflammation, decreasing the release of inflammatory cytokines, therefore promoting brain metastasis formation [24].

A link between amyloid proteins and cancer has also been established in melanoma where the amyloidogenic protein PMEL is currently used as a diagnostic marker [25]. Recently, PMEL has also been proposed as a prognostic marker for a poor overall survival of melanoma patients [26], but its role in melanoma development or progression is largely unknown.

In the present study, we show that amyloid aggregate accumulation is present in different tumor biopsies and that the amyloid-driven mechano-signaling is conserved at least in PDAC and melanoma. In addition, we unravel the molecular components of the amyloid fibril-induced signaling cascade that activates YAP. Further, we demonstrate that the amyloid-mechano-signaling plays a role in migration and invasion of cancer cells. Therefore, targeting this cascade can represent an alternative way to shut down YAP activation along with its detrimental effects.

## Materials and methods

### Cell lines

Three melanoma cell lines (IGR39, IGR37 and WM266.4) and six PDAC cell lines (BXPC3, ASPC-1, CAPAN-1, CAPAN-2, SU86.86, and PANC-1) were used in this study. Cells were plated in 10 cm dishes and cultured in Dulbecco’s modified Eagle medium (DMEM) (Lonza) supplemented with 10% fetal bovine serum South American (FBS-SA, Euroclone) and 2 mM L-Glutamine (Euroclone). Cells were incubated at 37 °C, 5% CO_2_. All of the cell lines were tested for mycoplasma, by mycoplasma PCR (Polymerase chain reaction) Test Kit.

### Treatments

IGR37 and WM266.4 cells were treated with DMSO (Euroclone) or 3I (Axon), 5 µM and 7 µM, respectively, for 48 h when they were at a confluency of 50%.

IGR37 and WM266.4 cells were treated with DMSO or 3I or 3I + rPMEL (10 µg/ml) for 48 h.

IGR39 cells were treated with 5 µg/ml rPMEL for 24 h.

IGR39 and IGR37 cells were treated with DMSO or 10 µM ROCKi (Y-27632) for 24 h.

BXPC3 cells were treated with 10 µM 3I and 0.5 µM Aβ40 peptides for 48 h.

IGR37 and BXPC3 were treated with 2 µM of Verteporfin for 48 h.

### Human tissues

PDAC tissues were obtained from patients undergoing surgical resection at the University Hospital Trust of Verona (Univr). Tissue specimens were collected under the protocol approved by the local IRB protocol number 1911 approved by the local Ethics Committee (Comitato Etico Azienda Ospedaliera Universitaria Integrata) to V.C. (Prot. n 61413, Prog 1911 on 19/09/2018). Written informed consent from the donors for research use of tissue in this study was obtained prior to acquisition of the specimen.

Colon and breast tissue sections for immunohistochemical analyses were retrieved from the archives of the Tumor Immunology Unit of the University of Palermo and the Pathology Unit of the Sant’Andrea Hospital of the Sapienza University, Rome, and were included in the 05/2018 study approved by the University of Palermo Institutional Review Board. Informed consent for histopathological studies was obtained upon collection; samples were obtained and handled according to the Declaration of Helsinki. Tissue sections were reviewed and analyzed by two expert pathologists (ADN, CT). All experiments were conducted in accordance with relevant guidelines and regulations. Tissues were fixed in 10% neutral buffered formalin and embedded in paraffin.

### Analysis of human biopsies

Formalin-fixed paraffin (PFA)-embedded tissues were sliced into serial 8-μm-thick sections and collected for immunohistochemical (IHC) staining.

For Proteostat aggresome detection, deparaffinized and rehydrated slides were fixed in 4% PFA for 15 min, incubated in Proteostat solution (1:1,000, Proteostat Aggresome Detection Kit, Enzo) for 3 min, and then destained in 1% acetic acid for 20 min at room temperature. To visualize the cell nuclei, human slides were counterstained with 4,6-diamidino-2-phenylindole (DAPI, Sigma–Aldrich), mounted with a phosphate-buffered saline/glycerol solution, and examined with confocal or widefield microscopy. Confocal microscopy was performed on a Leica TCS SP5 confocal laser scanning based on a Leica DMI 6000B inverted motorized microscope. The images were acquired with a HC FLUOTAR L 25X/NA0.95 VISIR water immersion objective using the 405 nm and the 488 nm laser lines. The software used for all acquisitions was Leica LAS AF.

### RNA extraction, RT–PCR and real‐time PCR

Total RNA was extracted using Maxwell RSC simply RNA (Promega, USA) according to manufacturer’s instructions, and RNA was quantified by nanodrop. 1 μg of total RNA was used for retro‐transcription using SuperScript™ VILO™cDNA Synthesis Kit (Invitrogen, USA). cDNA was diluted 1:10, and qPCR was performed using LightCycler® 480 SYBR Green I Master (Roche, Switzerland). The primer sequences are provided below. Expression data were normalized to the geometric mean of the housekeeping gene RPLP0 to control the variability in expression levels and were analyzed using the 2‐ΔΔCT method. Primers for qPCR:

**Table:** 

RPP0	Fw5′GTTGCTGGCCAATAAGGTG Rv5′GGGCTGGCACAGTGACTT
CYR61	Fw5′-AAACCCGGATTTGTGAGGT Rv5′GCTGCATTTCTTGCCCTTT
CTGF	Fw5′-GGGAAATGCTGCGAGGAG Rv5′-GCCAAACTGTCTTCCAGTC
Agrin	Fw5′- TTGTCGAGTACCTCAACGCT Rv5′- CAGGCTCAGTTCAAAGTCGT
LATS1	Fw5′- AAATGAGTTACCAAGATCCTCGAC Rv5′- CGGTTAACTGATTGCTGCAC
LATS2	Fw5′- AGCAAGAAATGGCCAAAGC Rv5′- GGTAGAGGATCTTCCGCATCT
BACE2	Fw5′- GCAACCATGAACTCAGCTATTAAGAA Rv5′- AGAAAGCGCCACCATCGA

### Immunofluorescence

4 × 10^4^ IGR37 cells were plated on sterile 13 mm coverslips and when the cells were at a confluency of 50%, they were treated with DMSO or 3I alone or together with rPMEL for 48 h.

Cells were fixed with PBS, 4% (wt/vol) PFA for 15 min at room temperature and washed three times with PBS. Permeabilization was performed with 0.3% Triton X-100 (Sigma–Aldrich) for 5 min, followed by three washes with PBS and 0.02% BSA.

Cells were incubated with specific YAP-antibodies (1:100, mouse monoclonal anti-YAP, Santa Cruz, sc-101199) diluted in PBS and 0.2% BSA for 1 h at room temperature and then washed three times with PBS and 0.2% BSA. Cells were incubated with PBS and 2% BSA for 15 min at room temperature and washed twice with PBS and 0.2% BSA.

Cells were incubated with the secondary antibodies (1:400, anti-mouse 488) diluted in PBS and 0.2% BSA for 1 h at room temperature, then washed three times with PBS and 0.2% BSA, incubated with PBS and 2% BSA for 15 min at room temperature and washed twice with PBS and 0.2% BSA.

Cells were washed with PBS, and stained with DAPI (1:5,000) (Sigma–Aldrich) for 5 min, followed by three washes with PBS.

Cells were then analyzed using confocal microscopy performed on a Leica TCS SP5, based on a Leica DMI 6000B inverted microscope equipped with motorized stage. The images were acquired with an HCX PL APO 63X/NA1.4 oil immersion objective using the 405 and 488 nm laser lines. The software used for all acquisitions was Leica LAS AF (on TCS SP5 system).

For Proteostat staining, cells were fixed in 4% PFA for 10 min and permeabilized with 0.3% Triton X for 5 min. After the treatment, cells were fixed with 4% (wt/vol) PFA, blocked with PBS-BSA (1% wt/vol), permeabilized with 0.2% Triton X-100 (Sigma–Aldrich) for 3 min, and incubated with Proteostat (1:1,000) or specific antibodies diluted in 0.2% BSA in PBS. Cells were then washed three times with PBS and stained with DAPI (1:5000, Sigma–Aldrich). Cells were analyzed using confocal microscopy performed on a Leica TCS SP5, based on a Leica DMI 6000B inverted microscope equipped with motorized stage. The images were acquired with an HCX PL APO 63X/NA1.4 oil immersion objective using the 405 and 488 nm laser lines. The software used for all acquisitions was Leica LAS AF (on TCS SP5 system).

### Circular Dichroism spectroscopy

CD spectra were acquired on a Jasco J-815 CD spectrometer at 37 °C, from 200 to 260 nm. Typical protein concentrations were 3 μM in 20 mM Na2HPO4/NaH2PO4 pH 7.2 and 150 mM NaF. Spectra were averaged over 4 scans and corrected by subtracting the buffer spectrum and smoothed.

### Electron microscopy analysis (Negative staining)

Renatured recombinant PMEL preparation was resuspended in TBS 1× at a final concentration of 50–500 µg/mL and adsorbed for 30 s on a 6 nm carbon layer subjected to glow discharging and previously deposited onto a 400-mesh copper electron microscopy grid. PMEL proteins adsorbed on the carbon surface were immediately negatively stained for one minute with a 2% solution (weight/volume) of uranyl acetate dissolved in deionized water and filtered. Excess of uranyl acetate staining solution was removed with filter paper and the grids dried at room temperature. Grids were analyzed at 120KV with an FEI Tecnai 12 G2 Biotwin transmission electron microscope (Bright Field). Pictures were acquired with a side-mounted Gatan Orius SC-1000 CCD camera controlled by the Digital Micrograph software. Pictures were analyzed and scale bars applied with the Image J software.

### Secretome preparation from cell cultures and digestion

Secretome were prepared as described in Matafora et al. [27].

### Secretome insoluble fraction preparation

250 μg of protein were extracted from the secretome in PBS 1-1% Triton X and subjected to ultracentrifugation at 100,000 × *g* for 1 h. After the centrifugation, the supernatant, corresponding to the soluble fraction, was removed and the pellet, corresponding to the insoluble fraction, was washed 3 times with PBS to remove the contaminant. Then the insoluble fraction was resuspended in PBS for dot blot or in 8 M Urea for proteomics analysis. For proteomics analysis we follow the same protocols as for the secretome preparation.

### Liquid chromatography and mass spectrometry analysis

1.5 μl of digested sample was injected onto an Exploris 480 mass spectrometer (Thermo Scientific) equipped with FAIMS device (Thermo Scientific).

Peptide separation was achieved with an 80 min gradient as following: from 0% of solvent B (80% acetonitrile, 0.1% formic acid) to 7% in 2 min. In 60 min, solvent B raised to 30%, and in 5 min to 60%. Finally, solvent B increased to 100% in 2 min and remained as such for 11 min. Solvent A was composed of 2% acetonitrile, 0.1% formic acid.

The flow rate was 0.20 μl/min on UHPLC Easy-nLC 1200 (Thermo Scientific) onto a 25-cm fused-silica emitter of 75 μm inner diameter (New Objective, Inc. Woburn, MA, USA), packed in-house with ReproSil-Pur C18-AQ 1.9 μm beads (Dr Maisch Gmbh, Ammerbuch, Germany) using a high-pressure bomb loader (Proxeon, Odense, Denmark).

The mass spectrometer was operated in ESI + in data-dependent acquisition (DDA) mode: charge state: 2–6, intensity threshold 5.0 × 10^3^, dynamic exclusion enabled (exclusion duration = 20 s), MS1 resolution = 60,000, MS1 automatic gain control target = 1 × 10^6^ (100%), MS1 maximum injection time = 100 ms, MS2 resolution = 15,000, MS2 automatic gain control target = 1 × 10^6^ (100%), MS2 maximum fill time = Auto, and MS2 HCD collision energy % = 28. For each cycle, one full MS1 scan range = 300–1,500 m/z was followed by 28 MS2 scans using an isolation window of 1.6 m/z.

The FAIMS device was operated with the following compensation voltages: −50 V and −70 V with a total carrier gas flow of 3.7 L/min at 100 °C for inner electrode, outer electrode 1 and 2.

### MS analysis and database search

Raw MS files were converted in MzxML files with FAIMS-MzxML generator (https://github.com/coongroup/FAIMS-MzXML-Generator) as they contain multiple CVs and were analyzed with MaxQuant (version 1.6.0.16), using Andromeda as searching engine.

MS/MS peak lists were searched against the UniProtKB Human complete proteome database (uniprot_cp_human_2020) in which trypsin specificity was used with up to two missed cleavages allowed. Searches were performed selecting alkylation of cysteine by carbamidomethylation as fixed modification, and oxidation of methionine, N-terminal acetylation and N-Deamination as variable modifications.

Mass tolerance was set to 5 ppm and 10 ppm for parent and fragment ions, respectively. A reverse decoy database was generated within Andromeda, and the false discovery rate (FDR) was set to <0.01 for peptide spectrum matches (PSMs). For identification, at least two peptide identifications per protein were required, of which at least one peptide had to be unique to the protein group.

### Western blot and dot blot assays

For Western blot analyses, proteins were extracted in buffer containing 8 M Urea, 100 mM Tris–HCl pH 8. Briefly, cell lysates (50 μg) were separated by SDS–PAGE using a precast polyacrylamide gel with a 4% to 12% gradient (Invitrogen). After the electrophoretic run, proteins were transferred onto a 0.22 μm nitrocellulose membrane, for dot blot we spotted the protein mix directly to nitrocellulose membrane (Amersham Protran, GE Healthcare) in wet conditions. The assembled sandwich was loaded in a Trans‐Blot Cell (Bio‐Rad) and immersed in 1× cold Tris‐Glycine transfer buffer with the addition of 20% methanol. The transfer was allowed overnight at constant voltage (30 V). Correct protein transfer was verified staining the membrane with Ponceau red (Sigma‐Aldrich) for few seconds. After washing the membrane with Tris‐buffered Saline‐Tween 20 (TBST, 1× TBS with 0.1% Tween‐20), non‐specific binding of antibodies was blocked by adding 5% low‐fat dry milk in TBST for 1 h at room temperature. The antibody used are anti-LATS1 (Cell Signaling, 1:1000, rabbit), anti-LATS2 (Cell Signaling, 1:1000, rabbit), anti-BACE2 (Sigma Prestige, 1:250, rabbit) and anti-OC (Merck, 1:1000, Rabbit), anti-YAP-antibodies (Santa Cruz, 1:200, mouse), anti-p-YAP (Ser127) (Cell Signaling, 1:1000, Rabbit).

### Recombinant PMEL (rMα) expression and purification

Recombinant PMEL was produced and purified as described in Matafora et al. [10].

### PMEL pull down

10 μg of recombinant purified PMEL amyloid fibrils were attached to 100 μL Ni-NTA beads 50% slurry, overnight at 4 °C. The day after, 100 μg of protein coming from CM of metastatic melanoma cells were added to beads coated with PMEL amyloid fibrils or Ni-NTA beads without PMEL amyloid fibrils as control for 1 h at room temperature. The beads were washed 3 times with washing buffer (50 mM Imidazole, 500 mm NaCl, 20 mM Tris–HCl) and then eluted directly in elution buffer (500 mM Imidazole, 500 mM NaCl, 20 mM Tris–HCl). Proteomics analysis were conducted as the secretome analysis with shorter gradient (45 min).

### LATS1/2 overexpression

For LATS1 overexpression pEGFP C3-LATS1 was used; for LATS2 overexpression pcDNA3.1-GST-hLATS2 plasmid was used; pcDNA3-EGFP was used as control for the transfection. IGR39 and IGR37 cells were transfected using ScreenFectA reagent according to manufacturer’s instruction. Briefly, two mix were prepared; for MixA 6 µL ScreenFectA were diluted in 120 µL of ScreenFect dilution buffer. For MixB 2 µg of pEGFP C3-Lats1 (LATS1OE), pcDNA3.1-GST-hLATS2 (LATS2 OE) or pcDNA3-EGFP (Control) plasmid DNA were diluted in 120 µL of ScreenFectA dilution buffer. MixA and MixB were then mixed together using rapidly slight pipette strokes and incubated for 20 min at room temperature to allow DNA/Lipofectamine complex formation. After that, 1260 µL fresh cell suspension at a concentration of 4 × 10^5^ cells/mL were added to complexes and gently mixed with pipette and plated in wells of 6wells/multiwell.

The day after, the cell culture medium was changed to avoid the lipofectamine toxic effects After 48 h, cells were collected to check the overexpression.

### Agrin and BACE2 silencing

IGR39 and IGR37 cells were transfected using ScreenFect siRNA reagent according to manufacturer’s instruction. Briefly, two mixtures were prepared; for MixA, 4 µL ScreenFect siRNA were diluted in 120 µL of ScreenFect dilution buffer, and for MixB, 10 nM of siRNA against non-coding region (siNC) or siRNA against Agrin (siAGRN) or BACE2 (siBACE2) were diluted in 120 µL of ScreenFectA dilution buffer. MixA and MixB were then mixed together using rapid, slight pipette strokes and incubated for 20 minutes at room temperature to allow siRNA/Lipofectamine complex formation. After that, 1260 µL fresh cell suspension at a concentration of 2 × 10^5^ cells/mL were added to the complexes and gently mixed with a pipette and plated in 6-well plates.

The following day, the cell culture medium was changed to avoid the toxic effects of lipofectamine. After 72 h for Agrin and after 24 h for BACE2, the cells were collected to assess the silencing and to perform downstream experiments. Sequences of siRNA used:

**Table:** 

siAGRN	CAUACGGCAACGAGUGUCAGCUGAA UUCAGCUGACACUCGUUGCCGUAUG
siBACE2	GAUUCUCGUUGACACUGGA UCCAGUGUCAACGAGAAUC
siNC	ACGUGACACGUUCGGAGAA UUCUCCGAACGUGUCACGU

### Wound healing assay

IGR39 cells were seeded at 80% confluency. After 24 h, the cells were treated with PMEL amyloid fibrils and Verteporfin. After another 12 h a wound was performed manually with a 200 μL pipette tip. Image acquisition was recorded by Leica AM TIRF MC system with 10× objective for 18 h.

IGR37, WM266.4 and BXPC3 cells were seeded at 80% confluency. After 24 h after were treated with DMSO, 3I alone, 3I with PMEL or Aβ40 and in combination with Verteporfin. 12 h after the treatment, a wound was performed manually with a 200 μL pipette tip IGR37 cells were imaged for 48 h, whereas WM266.4 and BXPC3 cells were imaged for 24 h.

### Immunofluorescence of spheroids culture

Spheroids were grown and treated as described in the main text. After treatment, single spheroid was collected in 15 mL tube and the media was removed. Then, the spheroids were washed twice in PBS 1× and resuspended in PFA 4% for 20 min to allow fixation of the sample. After fixation, the spheroids were washed three times with PBS 1× and blocked with BSA2% for 2 h. Then, Proteostat at 1:1000 final concentration was added for 3 h at room temperature and the sample were washed 3 times in PBS 1×. After, DAPI was added at 1:5000 final concentration for 30 min at room temperature. Samples were spotted on glass coverslip, mounted with glycerol and the images were acquired used a were analyzed using confocal microscopy performed on a Leica TCS SP5, based on a Leica DMI 6000B inverted microscope equipped with motorized stage. The images were acquired with an HCX PL APO 63X/NA1.4 oil immersion objective using the 405 and 488 nm laser lines. The software used for all acquisitions was Leica LAS AF (on TCS SP5 system).

### Spheroid invasion assay

Tumor spheroids were formed by suspending 2000 cells (WM266.4) in 100 μL cell culture medium per well in a 96-well ultra-low attachment U-bottom plate (Corning) and incubated (37 °C and 5% CO2) for 72 h. At this point, spheroids reached a diameter of about 500 μm. Then, the spheroids were embedded in 60% Matrigel^TM^ by aspirating 40 μL medium and adding 60 μL ice-cold Matrigel. The plates were incubated (37 °C and 5% CO2) for 1 h to solidify completely before adding 100 μL cell culture medium to each well. Microphotographs were taken at 0 and 48 h using an optical microscope.

### Apoptosis assay

Annexin V-fluorescein isothiocyanate (FITC)/propidium iodide (PI) double staining kit (FITC-conjugated Annexin V) (eBioscience, USA) was used to label apoptosis cells. Briefly, 1 × 10^6^ cells were resuspended in 0.5 ml staining binding buffer, and then, Annexin V-FITC (5 μl) and PI (1 μl) were added to the cells, respectively. Cells were stained for 15 min at room temperature and subjected to flow cytometry analysis.

### Cell cycle analysis

1 × 10^6^ cells were fixed with 70% ethanol. Cells were then treated with 2 mg/mL (final concentration) RNAseA (Sigma) in Tris–HCl 50 mM pH 7.5 for at least 1 h at 37 C. Later, cells were stained with 50 mg/ml Propidium Iodide (PI) (Sigma) in Buffer solution (180 mM Tris–HCl pH 7.5, 190 mM NaCl, 70 mM MgCl2) overnight. PI-stained cells were subjected to flow cytometry analysis by using Becton Dickinson FACScan for FL2H fluorescence.

### MTT cell viability assay

To perform 3-(4,5-dimethylthiazol-2-yl)-2,5-diphenyltetrazolium bromide (MTT; Sigma) cell viability assay, melanoma cells were seeded in 96-well plates and were treated with 3I, rPMEL amyloid fibrils or combination of the two, as indicated in the text. At the end of the experiments, the cell cultures were supplemented with 150 μl of 0.5 mg/ml MTT assay and incubated for an additional 4 h. Then, equal volume of solubilizing solution (dimethyl sulfoxide 40%, SDS 10% and acetic acid 2%) was added to the cell culture to dissolve the formazan crystals and incubated for 10 min at room temperature. The absorbance rate of the cell culture was detected at 570 nm by using a Microplate Reader (Bio-Rad, Hercules, CA, USA).

### Statistical analysis

All statistical analysis of the experimental data was performed using the GraphPad Prism version 9.5.0 for macOS, GraphPad Software, San Diego, CA, USA, www.graphpad.com software. All the experiments were performed using at least 3 biological replicates. Statistical significance for each experiment is marked in the form of asterisks (*) along with the calculated *p*-value for experiments are shown.

## Results

### Amyloid aggregates accumulate in several cancer tissues

BACE2 and its targets are overexpressed in several solid tumors [28]. BACE2 was firstly studied for its role in the formation of the amyloid peptides involved in the pathogenesis of Alzheimer disease [29]. More recently, our and other research groups have addressed the involvement of BACE2 in cancer establishment and progression, in particular in melanoma and pancreatic cancer, where the processing of amyloidogenic proteins has been shown to increase cancer growth and metastasis formation [23, 24, 28].

**Fig. 1:**
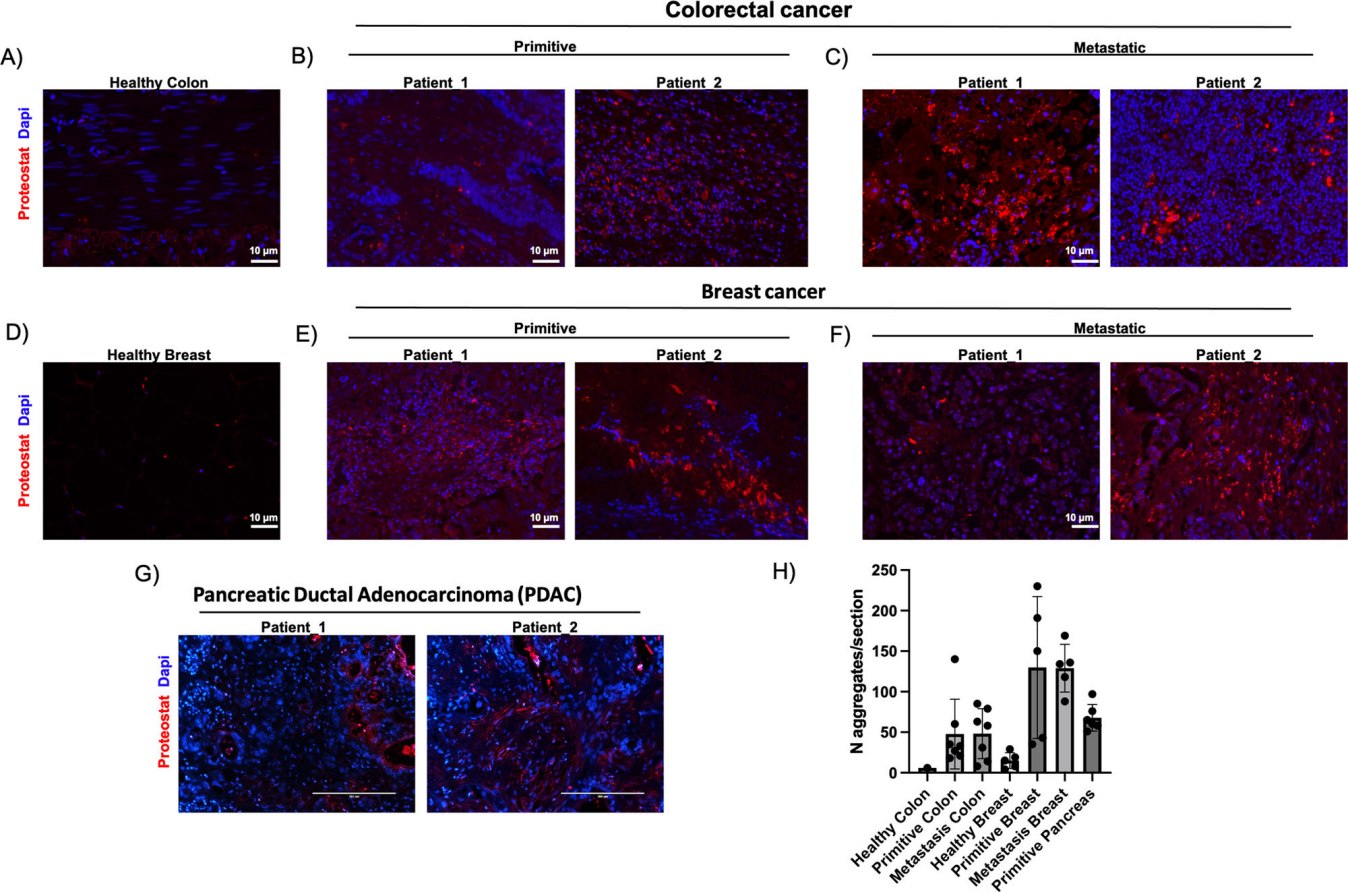
Amyloid aggregates accumulate in cancer tissues. Immunofluorescence images of human biopsies stained for amyloid aggregates (Proteostat in red) and nuclei (DAPI in blue). **A** Healthy colon tissue, **B** primitive colorectal cancer, **C** metastatic colorectal cancer; scale bar: 10 μm. **D** Healthy breast tissue, **E** primitive breast cancer, **F** metastatic breast cancer; scale bar: 10 μm, and **G** pancreatic ductal adenocarcinoma (PDAC), scale bar: 200 nm. **H** Quantitation of Proteostat signal per tissue section.

Formalin-fixed paraffin-embedded (FFPE) specimens collected from healthy, primitive colorectal and breast cancer, matched metastasis and PDAC lesions were stained with Proteostat, an amyloidophilic dye that emits fluorescence when it reacts with β-sheets, which is a common feature of amyloid aggregates. The sections were analyzed by high-resolution, large scale confocal imaging. Both in primitive and metastatic tissues from colorectal cancer, we detected high representation of protein aggregates compared to healthy counterpart (Fig. 1A–C). Also in breast cancer tissues we noticed the same kind of enrichment in tumor samples compared to the healthy tissue (Fig. 1D–F). Further, we analyzed primitive PDAC lesion, which resulted to be highly positive for Proteostat staining (Fig. 1G). These data support the hypothesis that the presence of amyloid aggregates is spread among several types of cancer tissues and in different stage of the disease (Fig. 1H).

### Amyloid aggregates drive YAP activation in PDAC

**Fig. 2:**
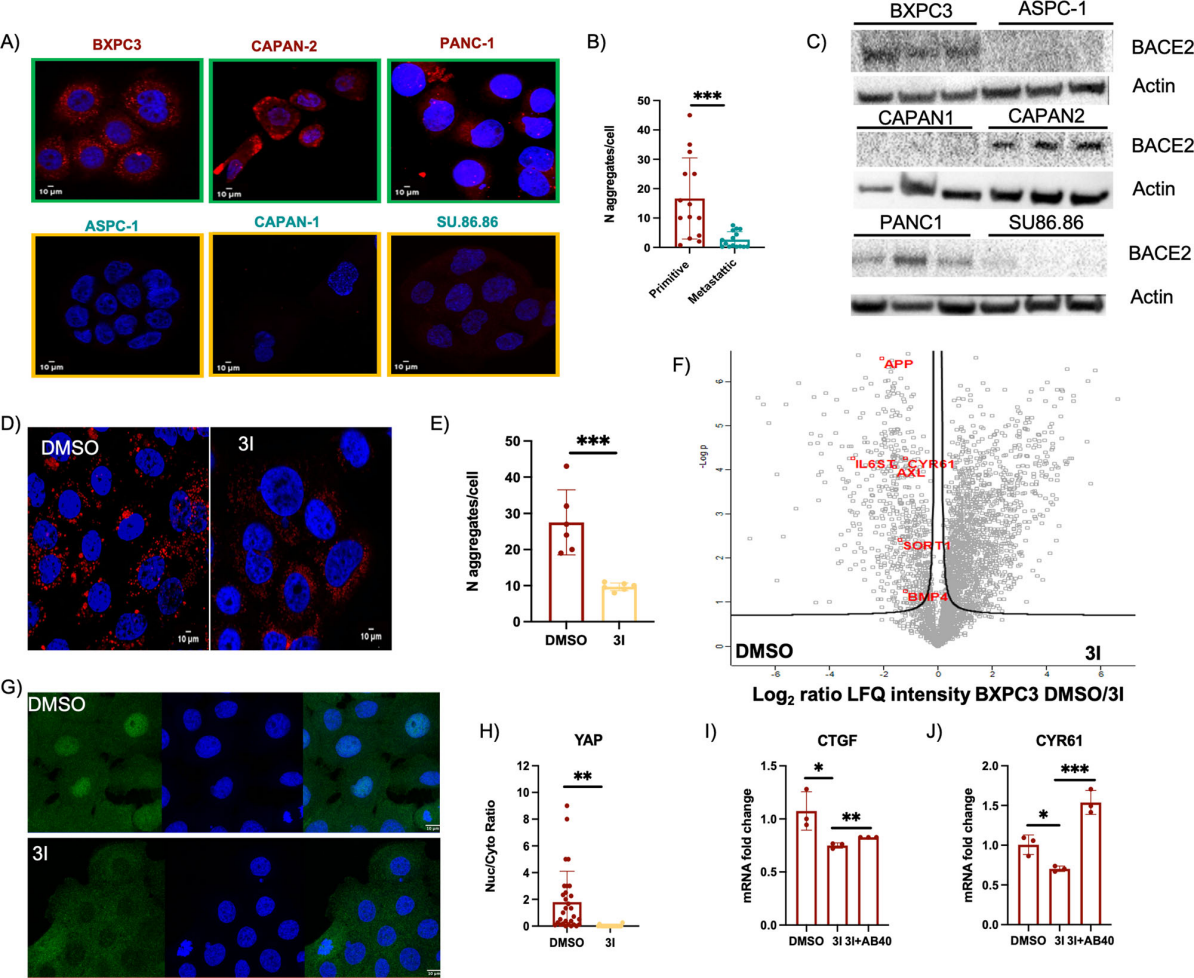
Amyloid aggregates accumulate in primitive PDAC cell lines driving YAP activation. **A** Confocal fluorescence images of Proteostat (1:1,000, red) and DAPI staining (blue) of primitive (BXPC3, CAPAN-2, PANC-1) and metastatic (ASPC-1, CAPAN-1, SU.86.86) PDAC cell lines. Scale bar: 10 μm. **B** Quantification of amyloid aggregates in primitive and metastatic PDAC cell lines by immunofluorescence analysis using Fiji software. *N* = 14. *T*-test, ***P < 0.001. Data are presented as mean ± SD. **C** Western blot of BACE2 expression in primitive (BXPC3, CAPAN-2, PANC-1) and metastatic (ASPC-1, CAPAN-1, SU.86.86) PDAC cell lines. Actin was used as loading control. *N* = 3 biological replicates. **D** Confocal fluorescence images of Proteostat (1:1,000, red) and DAPI staining (blue) of BXPC3 treated with DMSO or 3I. Scale bar: 10 μm. **E** Quantification of amyloid aggregates in BXPC3 cell lines, treated with DMSO or 3I for 48 h, by immunofluorescence analysis using Fiji software. *N* = 5 biological replicates. *T*-test, ***P < 0.001. Data are presented as mean ± SD. **F** Volcano plot of the proteins secreted by BXPC3 cells treated with DMSO or 3I. **G** Confocal fluorescence images of anti-YAP antibody (green) and DAPI staining (blue) in BXPC3 upon treatment with DMSO or 3I. Scale bar: 10 μm. **H** Quantification, by immunofluorescence analysis, of YAP nuclear/cytosol ratio. *N* = 20 replicates. *T*-test analysis, **P < 0.01. Data are presented as mean ± SD. **I** CTGF mRNA level measured by real-time PCR in BXPC3 treated with DMSO or 3I, or 3I and recombinant Aβ40 (0.5 μM), *N* = 3 biological replicates. *T*-test analysis *P < 0.05, **P < 0.01. Data are presented as mean ± SD. **J** CYR61 mRNA level measured by real-time PCR in BXPC3 treated with DMSO or 3I, or 3I and recombinant Aβ40 (0.5 μM). *N* = 3 biological replicates. *T*-test analysis *P < 0.05, ***P < 0.001. Data are presented as mean ± SD.

Since we detected the presence of amyloid aggregates in human biopsies of pancreatic cancer, we then analyzed the presence of amyloid aggregates in a panel of different PDAC cell lines. The analysis showed that primitive PDAC cell lines (BXPC3, PANC-1, and CAPAN-2) produce amyloid aggregates, while metastatic PDAC cell lines (ASPC-1, CAPAN-1, and SU86.86) were negative for Proteostat staining (Fig. 2A, B). Coherently, we found that only primitive cells express BACE2, the rate-limiting enzyme for the formation of amyloid aggregates (Fig. 2C). Indeed, as observed in melanoma cells [10], both pharmacological inhibition and genetic knockdown (Fig. S1A) of BACE2 reduce the level of amyloid aggregates in BXPC3 (Fig. 2D, E; Fig. S1B, C). To verify that protein aggregates mediate YAP activation, as previously observed in melanoma [10], we analyzed the secretome of BXPC3 cells treated with BACE2 inhibitor (3I). Indeed, secretome analysis showed that BACE2 inhibition, on the one hand, decreases the shedding of known BACE2 targets such as SORT1, IL6ST [30] and APP, and on the other, downregulates different YAP target genes such as CYR61, AXL, and BMP4 (Fig. 2F, Table S1). These data indicate an inhibition of YAP transcriptional activity as demonstrated by the observation that, upon BACE2 inhibition, YAP mainly localizes in the cytosol (Fig. 2G, H). To ensure that YAP deactivation is dependent on BACE2 activity and not due to off targets of the drug, we knocked down BACE2 in BXPC3 and we measured the expression of CTGF and CYR61, two YAP target genes, which resulted downregulated (Fig. S1D, E) similarly to what has been observed by pharmacological inhibition. Further, to demonstrate that YAP deactivation upon BACE2 inhibition depends on amyloid aggregates production, we treated BXPC3 cells with Aβ40 peptide and analyzed the expression level of CTGF and CYR61. Since we identified APP as a target of BACE2 in the BXPC3 secretome (Fig. 2F, Table S1), we reasoned that the Aβ40 peptide, a product of the proteolytic activity of BACE2 on APP and a component of amyloid plaques, could mimic the BACE2-dependent amyloid aggregates produced by BXPC3. As shown in Fig. 2I, J, the administration of BACE2 inhibitor decreases the expression of both CTGF and CYR61, while the concomitant administration of Aβ40 rescues the expression of the two YAP target genes. These data demonstrate that also in PDAC cells amyloid aggregates can activate mechanotransduction, thus inducing both YAP nuclear translocation and transcriptional activity.

### Agrin is a component of the extracellular amyloid-rich compartment and interacts with PMEL amyloid fibrils

**Fig. 3:**
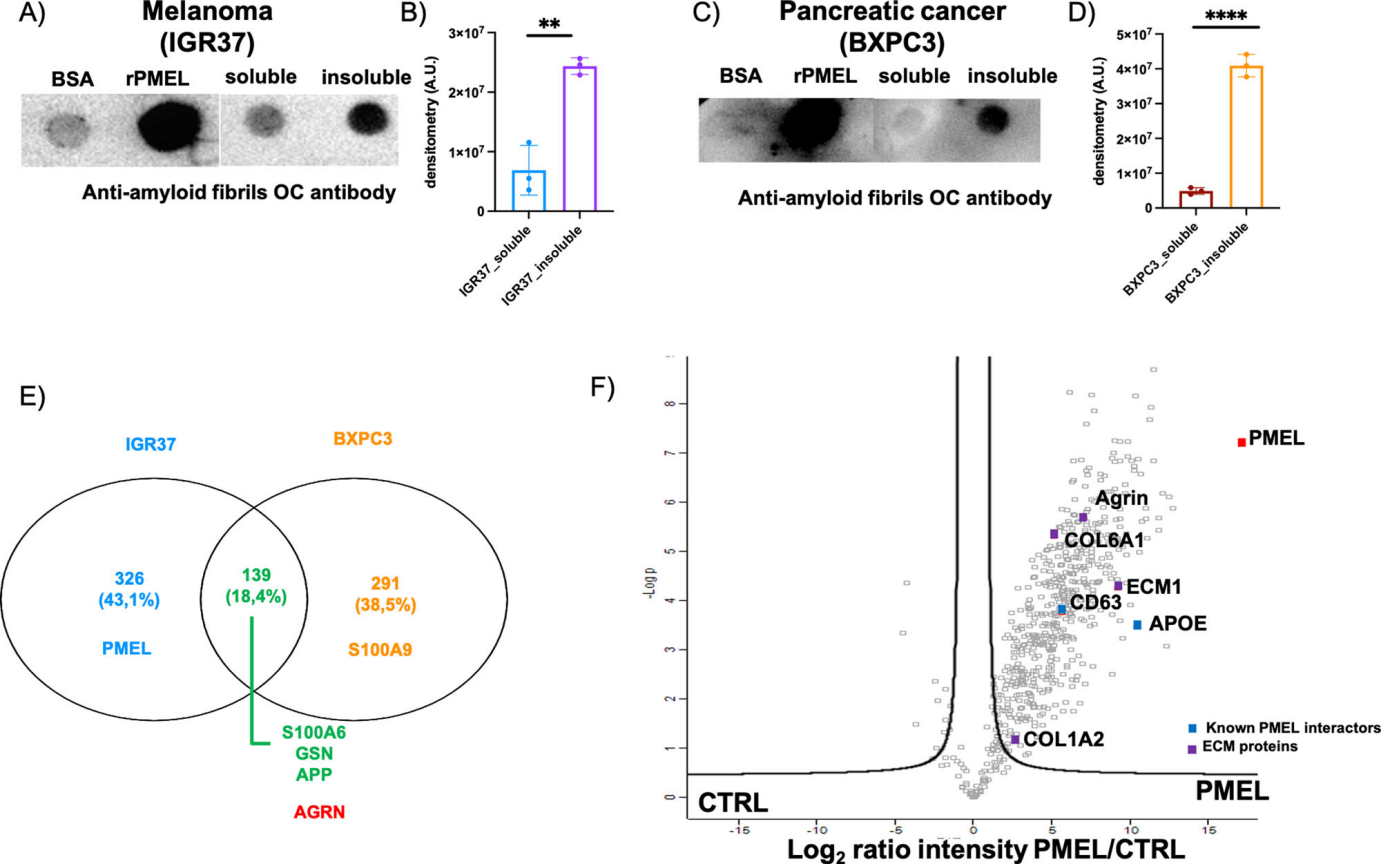
Agrin is enriched in secreted amyloid-rich compartments and interacts with PMEL amyloid fibrils. **A** Dot Blot of soluble and insoluble fractions of melanoma (IGR37) secretome probed with OC antibody. BSA: negative control. rPMEL: positive control. **B** Quantification of OC signal in the soluble and insoluble fractions of melanoma secretome. *N* = 3 biological replicates. *T*-test analysis, **P < 0.01. Data are presented as mean ± SD. **C** Dot Blot of soluble and insoluble fractions of pancreatic cancer (BXPC3) secretome probed with OC antibody. BSA: negative control. rPMEL: positive control. **D** Quantification of OC signal in the soluble and insoluble fractions of pancreatic cancer secretome. *N* = 3 biological replicates. *T*-test analysis, ****P < 0.0001. Data are presented as mean ± SD. **E** Venn diagram of the identified proteins through LC-MS/MS in the insoluble fraction of melanoma (IGR37) and pancreatic cancer (BXPC3) insoluble secretome. **F** Volcano plot showing the enrichment of the bait 6xHis-PMEL (red square) and its secreted interactors. Blue square: known PMEL fibrils interactors. Violet square: ECM proteins.

Amyloid species can form monomers and oligomers, soluble in detergent-rich solutions, and protofibrils and fibrils, which are inherently insoluble [31]. As BACE2 mainly participates in the formation of amyloid fibrils, we focused on fibrillar amyloid aggregates. Hence, we specifically isolated the secretome insoluble fraction from both melanoma and PDAC cells to elucidate the molecular composition of the secreted amyloid aggregates. The soluble and insoluble fractions were spotted on a nitrocellulose membrane and probed with a conformational specific antibody (OC), which is able to distinguish monomers from oligomers, and reacts only with the fibrillar form [32]. As expected, the secretome insoluble fraction of the metastatic melanoma cells (IGR37) and primitive PDAC cells (BXPC3) showed high reactivity to the OC antibody, demonstrating that the amyloids present in the extracellular space are in a fibrillar form (Fig. 3A–D). We then analyzed the protein composition of the insoluble fraction using LC-MS/MS for protein identification. 139 proteins were specifically enriched in the insoluble fraction of secreted proteins from both metastatic melanoma and PDAC cells (Fig. 3E). Among the proteins identified in the insoluble secretome, we found several amyloidogenic proteins such as APP, GSN and S100A6 [33]. In addition, we identified PMEL exclusively in melanoma cells and S100A9 exclusively in PDAC cells. Interestingly, besides amyloidogenic proteins, we identified Agrin in both metastatic melanoma and PDAC cells (Fig. 3E, Table S2), suggesting that this protein could be part of the amyloid plaques secreted by cancer cells. We found particularly interesting the presence of Agrin, as this glycoprotein is a component of the glycocalyx [34, 35]. The glycocalyx is a layer of glycoprotein and proteoglycan that cover the surface of mammalian cells [36]. It is reported that the glycocalyx has mechanosensing properties [37], perceiving the blood flow shear stress and activating mechanotransduction in endothelial cells [38].

Moreover, it has been demonstrated that Agrin can perceive the ECM stiffness and cause YAP activation [39]. In addition, Agrin accumulates in the brain of individuals affected by Alzheimer’s disease, specifically at the level of amyloid plaques [40], interacting with Aβ peptides [41]. These observations and our data showing the presence of Agrin in the insoluble secretome fraction led us to think that Agrin could represent the mechanosensor of cancer-secreted amyloid fibrils.

In order to identify amyloid fibril interactors, we produced His-tagged PMEL fibrils (rPMEL), which are mainly composed of β-sheets (Fig. S2A), show a fibrillar twisted form when analyzed by electron microscopy (Fig. S2B), and display a positive reaction to the amyloidophilic dye Proteostat (Fig. S2C). rPMEL fibrils were used as bait in pull-down experiment of metastatic melanoma secretome. LC-MS/MS analysis of the purified proteins showed that PMEL amyloid fibrils interact with already known interactors (APOE and CD63) [42], different ECM proteins (COL6A1, COL2A1, FN and ECM1) and Agrin (Fig. 3F, Table S3). These data strengthen the hypothesis that Agrin is the mechanosensor of amyloid fibrils.

### Agrin is necessary for the activation of PMEL amyloid fibril-induced mechanotransduction

**Fig. 4:**
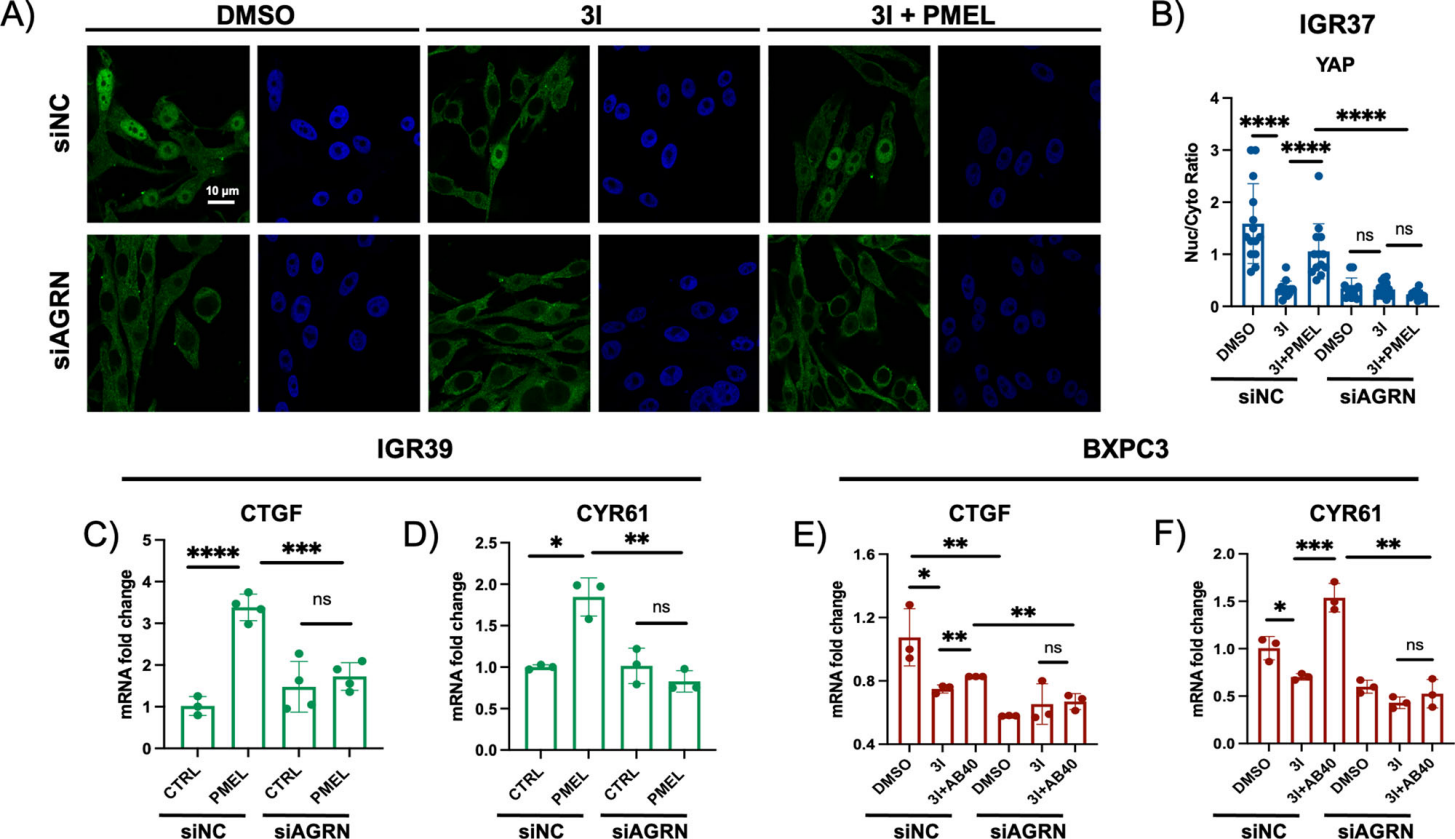
Agrin is necessary for amyloid-driven mechanoresponse. **A** Confocal fluorescence images of anti-YAP antibody (green) and DAPI staining (blue) in wild type (WT) IGR37 (siNC) cells upon treatment with 3I alone or in combination with rPMEL amyloid fibrils for 48 h, and of IGR37 Agrin KD cells (siAGRN) upon treatment with 3I alone or in combination with rPMEL amyloid fibrils for 48 h. Scale bar: 10 µm. **B** Nuclear/cytosol ratio of WT IGR37 (siNC) cells upon treatment with 3I alone or in combination with rPMEL amyloid fibrils for 48 h, and of IGR37 Agrin KD cells (siAGRN) upon treatment with 3I alone or in combination with rPMEL amyloid fibrils for 48 h. *N* = 15 replicates. *T*-test analysis, ****P < 0.0001. Data are presented as mean ± SD. **C** CTGF mRNA fold change in IGR39 WT (siNC) or Agrin KD cells (siAGRN) treated or not with rPMEL amyloid fibrils. *N* = 4 biological replicates. *T*-test analysis: ***P < 0.001, ****P < 0.0001. Data are presented as mean ± SD. **D** CYR61 mRNA fold change in IGR39 WT (siNC) or Agrin KD cells (siAGRN) treated or not with rPMEL amyloid fibrils. *N* = 3 biological replicates. *T*-test analysis: *P < 0.05, **P < 0.01. Data are presented as mean ± SD. **E** CTGF mRNA fold change in WT BXPC3 (siNC) or Agrin KD cells (siAGRN) treated with DMSO or 3I, or 3I and recombinant Aβ40. *N* = 3 biological replicates. *T*-test analysis: *P < 0.05, **P < 0.01. Data are presented as mean ± SD. **F** CYR61 mRNA fold change in WT BXPC3 (siNC) or Agrin KD cells (siAGRN) treated with DMSO or 3I, or 3I and recombinant Aβ40. *N* = 3 biological replicates. *T*-test analysis: *P < 0.05, **P < 0.01, ***P < 0.001. Data are presented as mean ± SD.

In order to verify that Agrin is involved in amyloid-driven mechanotransduction, we analyzed the YAP nuclear translocation in Agrin knockdown (KD) cells (Fig. S3A). We observed that, in IGR37 metastatic melanoma cells, the inhibition of BACE2 (3I), which decreases amyloid maturation and secretion [10, 28], decreases also YAP nuclear localization (Fig. 4A). However, the administration of rPMEL amyloid fibrils restores YAP nuclear localization (Fig. 4A). Conversely, in Agrin KD cells, neither the treatment with 3I nor the rescue with rPMEL amyloid fibrils affects YAP cellular localization (Fig. 4B), indicating that Agrin is necessary for amyloid-driven mechanoresponse.

Additionally, Agrin-proficient IGR37 cells treated with the BACE2 inhibitor 3I phenocopy the untreated Agrin-deficient cells (Fig. 4A, B), suggesting that removal of the fibrils or the sensor of the fibrils decreases YAP nuclear level in a similar fashion.

In addition, the administration of exogenous PMEL amyloid fibrils did not increase the expression of YAP target genes upon Agrin silencing (Fig. S3B) in amyloid-deficient melanoma cells (IGR39) (Fig. 4C, D). To verify that Agrin is involved in the signaling cascade and not in the processing of amyloid fibrils, we performed Proteostat staining in IGR37 upon Agrin knockdown. The data show that Agrin does not impact on the production of amyloid aggregates (Fig. S3C, D), while it is necessary to induce YAP transcriptional activation. These data strengthen the hypothesis that Agrin is a crucial molecule that promotes YAP nuclear translocation induced by amyloid fibrils.

Further, Agrin KD in BXPC3 PDAC cells (Fig. S3E) abrogates the effects of BACE2 inhibition and Aβ40 administration on the expression of YAP target genes, CTGF and CYR61 (Fig. 4E, F), demonstrating that this mechanism is not restricted to melanoma or PMEL amyloid fibrils but it is conserved in pancreatic cancer and can be driven by different types of amyloids, such as Aβ40 amyloids.

### PMEL amyloid fibrils activate YAP in a ROCK-independent, LATS1-dependent fashion

Downstream of Agrin, YAP can be activated by ROCK-dependent actin cytoskeleton remodeling and HIPPO pathway deactivation, which mostly rely on the deactivation of LATS1 and LATS2, core kinase of the HIPPO pathway, that through phosphorylation of YAP at Ser127 allow its cytosolic retention [15].

**Fig. 5:**
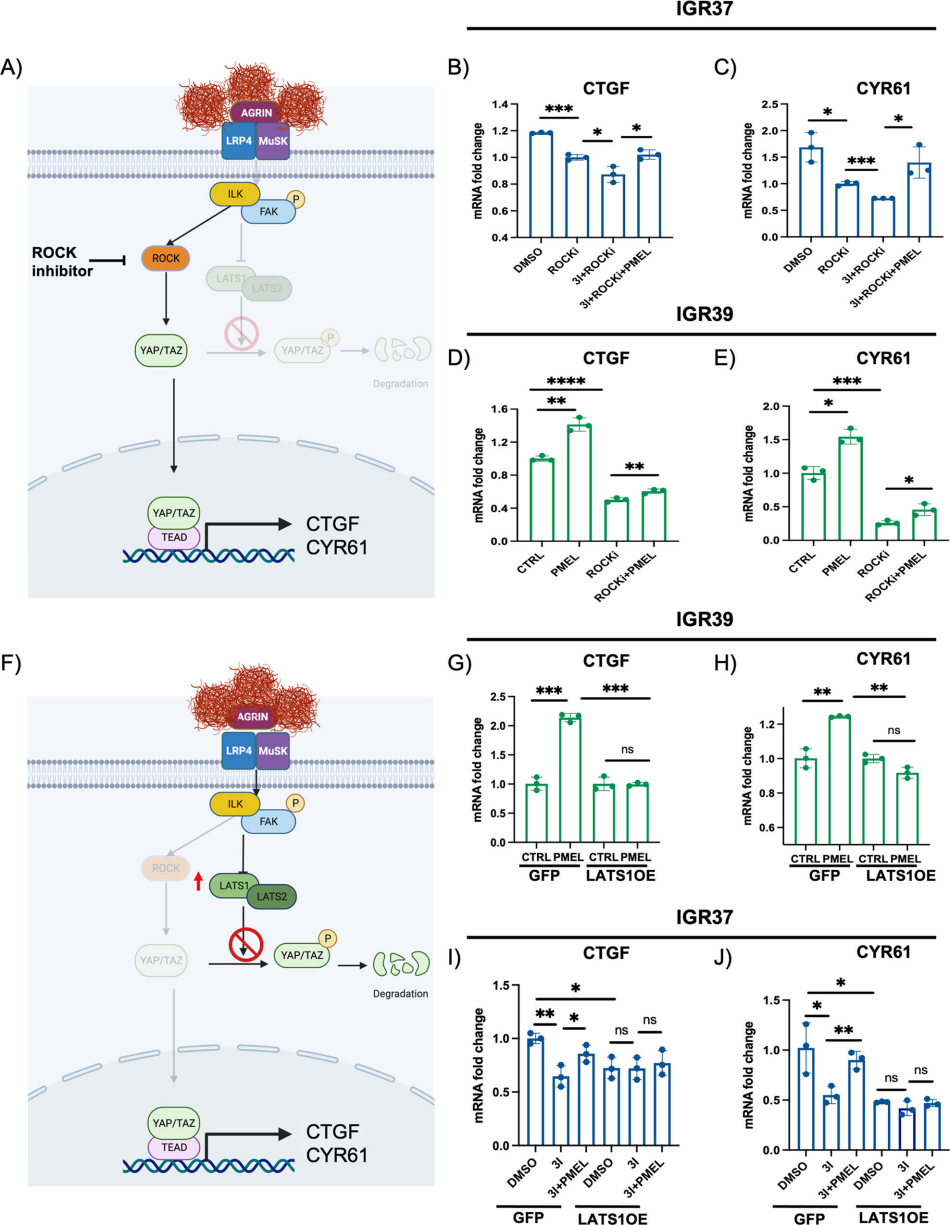
LATS1 blocks amyloid-driven mechanotransduction. **A** Representative cartoon of ROCK inhibition in the analyzed signaling pathway. **B** CTGF mRNA level in IGR37 cells treated with DMSO, ROCK inhibitor, ROCK inhibitor and 3I, or ROCK inhibitor, 3I and rPMEL amyloid fibrils. *T*-test analysis: *P < 0.05, ***P < 0.001. Data are presented as mean ± SD. **C** CYR61 mRNA level in IGR37 cells treated with DMSO, ROCK inhibitor, ROCK inhibitor and 3I, or ROCK inhibitor, 3I and rPMEL amyloid fibrils. *T*-test analysis: *P < 0.05, ***P < 0.001. Data are presented as mean ± SD. **D** CTGF mRNA level in IGR39 cells treated with rPMEL amyloid fibrils, ROCK inhibitor, or ROCK inhibitor and rPMEL amyloid fibrils. T-test analysis: **P < 0.01, ****P < 0.0001. Data are presented as mean ± SD. **E** CYR61 mRNA level in IGR39 cells treated with rPMEL amyloid fibrils, ROCK inhibitor, or ROCK inhibitor and rPMEL amyloid fibrils. *T*-test analysis: *P < 0.05, ***P < 0.001. Data are presented as mean ± SD. **F** Representative cartoon of LATS1 overexpression in the analyzed signaling pathway. **G** CTGF mRNA level in IGR37 WT (GFP) and IGR37 cells overexpressing LATS1 (LATS1OE) treated with DMSO or 3I, or 3I and rPMEL amyloid fibrils (3 l+PMEL). *T*-test analysis: ^ns^ *P* > 0.05, *P < 0.05, **P < 0.01. Data are presented as mean ± SD. **H** CYR61 mRNA level in IGR37 WT (GFP) and IGR37 cells overexpressing LATS1 (LATS1OE) treated with DMSO or 3I, or 3I and rPMEL amyloid fibrils (3 l+PMEL). *T*-test analysis: ^ns^ *P* > 0.05, *P < 0.05, **P < 0.01. Data are presented as mean ± SD. **I** CTGF mRNA level in IGR39 WT (GFP) or LATS1 overexpressing cells (LATS1OE) treated or not with rPMEL amyloid fibrils. ^ns^ *P* > 0.05, ***P < 0.001. Data are presented as mean ± SD. **J** CYR61 mRNA level in IGR39 WT (GFP) or LATS1 overexpressing cells (LATS1OE) treated or not with rPMEL amyloid fibrils. ^ns^ *P* > 0.05, **P < 0.01. Data are presented as mean ± SD.

To unravel the main players of the amyloid-driven mechanoresponse, ROCK was inhibited by using Y-27632 in IGR37 metastatic melanoma cells (Fig. 5A). First, we checked if ROCK inhibition affects amyloid deposition. Hence, we stained IGR37, treated with Y-27632, with Proteostat, and we could not detect any difference in amyloid processing (Fig. S4A, B). Then, we looked at the effects of ROCK inhibition on YAP activation. IGR37 treated with Y-27632 showed a decreased YAP activation in basal conditions, but the concomitant BACE2 inhibition further reduced YAP target genes expression (Fig. 5B, C). The administration of rPMEL amyloid fibrils in BACE2-ROCK inhibited cells restored the expression of both CTGF and CYR61 at a level comparable to cells treated with ROCK inhibitor alone (Fig. 5B, C). Consistently, in amyloid-deficient melanoma cells, ROCK inhibition decreased the level of YAP activation, but the administration of rPMEL amyloid fibrils significantly increased the expression of both CTGF and CYR61 in ROCK-deficient cells (Fig. 5D, E). These data indicate that PMEL amyloid fibril-driven mechanotransduction poorly relies on ROCK activity. Therefore, we decided to investigate the involvement of the HIPPO pathway. Thus, we overexpressed LATS1 in the amyloid-deficient melanoma cells IGR39 (Fig. S4C), we administered recombinant PMEL and we checked the level of LATS1 and p-YAP (Ser127), a marker of YAP cytosolic retention. Western blot analysis (Fig. S4D) showed that, upon rPMEL amyloid fibrils administration YAP, LATS1 and p-YAP were downregulated, indicating that the signaling pathway that leads to YAP nuclear localization is active. Instead, in LATS1 overexpressing cells, the administration of rPMEL does not abrogate YAP phosphorylation (Fig. S4E, F), impeding its nuclear translocation and transcriptional activity as measured by target genes expression (Fig. 5G, H).

We then overexpressed LATS1 in the amyloid proficient IGR37 cells (Fig. S4G, H) and verified that this perturbation does not alter amyloid aggregates formation (Fig S4I, J). Instead, we noticed that in IGR37 metastatic melanoma cells overexpressing LATS1, BACE2 inhibition, and rPMEL fibril administration have no effects on YAP activation (Fig. 5I, J). Interestingly, the overexpression of LATS1 phenocopies the level of YAP activation registered in control cells treated with 3I, suggesting that eliminating the mechanical stimulus exerted by fibrils or the molecules able to transduce the signal produce the same degree of YAP activation.

Further, the overexpression of LATS2 in amyloid-deficient melanoma cells (Fig. S4K, L) does not block the effects of rPMEL amyloid fibrils on CTGF expression (Fig. S4M), indicating that increasing the level of LATS2 is not sufficient to shut down mechanotransduction exerted by PMEL amyloid fibrils. These results define LATS1 as the core kinase involved in amyloid-driven-mechanoresponse.

### Amyloid fibrils increase migration and invasion of cancer cells

**Fig. 6:**
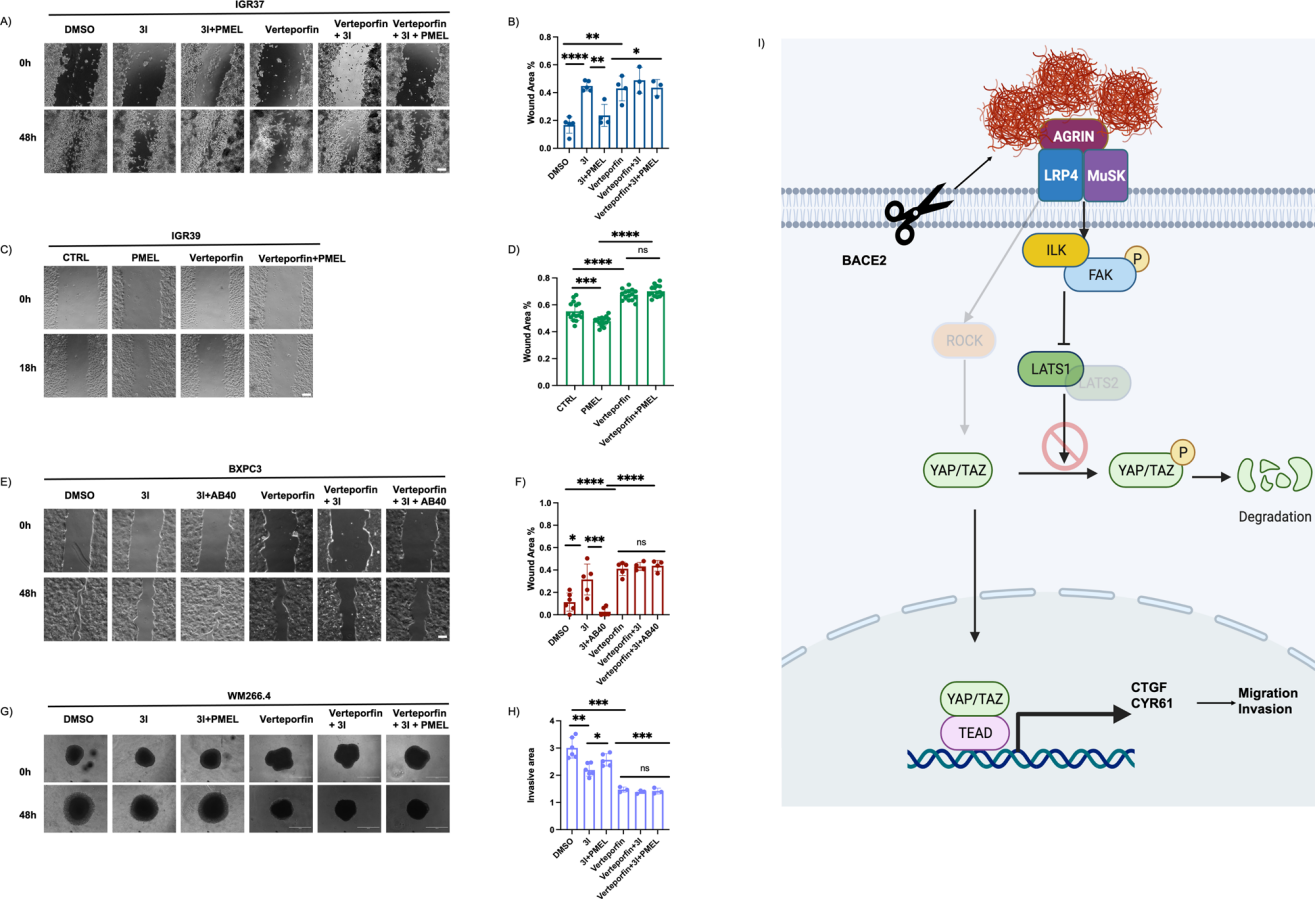
Amyloid fibrils increase migration and invasion capacity of cancer cells. **A** Representative micrographs of IGR37 cells treated with DMSO or, 3I, 3I plus recombinant PMEL amyloid fibrils (3I + PMEL), Verteporfin, Verteporfin plus 3I and Verteporfin plus 3I and recombinant PMEL amyloid fibrils in a wound healing assay at 0 and 48 h. Scale Bar 100 μm. **B** Box Plot of wound area for IGR37 cells treated with DMSO or, 3I, 3I plus recombinant PMEL amyloid fibrils (3I + PMEL), Verteporfin, Verteporfin plus 3I and Verteporfin plus 3I and recombinant PMEL amyloid fibrils at 48 h. *N* = 5 replicates. *T*-test analysis: *P < 0.05, **P < 0.01, ****P < 0.0001. Data are presented as mean ± SD. **C** Representative micrographs of IGR39 cells treated (PMEL) or not (CTRL) with recombinant PMEL amyloid fibrils alone or in combination with Verteporfin in a wound healing assay at 0 and 18 h. Scale Bar 100 μm. **D** Box Plot of wound area for IGR39 cells treated (PMEL) or not (CTRL) with recombinant PMEL amyloid fibrils or in combination with Verteporfin at 18 h. *N* = 19 replicates. *T*-test analysis: ***P < 0.001, ****P < 0.0001. Data are presented as mean ± SD. **E** Representative micrographs of BXPC3 cells treated with DMSO or, 3I, 3I plus Aβ40 Verteporfin, Verteporfin plus 3I, and Verteporfin plus 3I and Aβ40 in a wound healing assay at 0 and 48 h. Scale Bar 100 μm. **F** Box Plot of wound area for BXPC3 cells treated with DMSO or, 3I, 3I plus Aβ40 Verteporfin, Verteporfin plus 3I, and Verteporfin plus 3I and Aβ40 in a wound healing assay at 0 and 48 h. *N* = 4 replicates *T*-test analysis: *P < 0.05, ***P < 0.001, ****P < 0.0001. Data are presented as mean ± SD. **G** Representative images of WM266.4 spheroid Matrigel invasion assay at 0 and 48 h treated with DMSO or, 3I, 3I plus recombinant PMEL amyloid fibrils (3I + PMEL), Verteporfin, Verteporfin plus 3I and Verteporfin plus 3I and recombinant PMEL amyloid fibrils. Scale Bar 1000 μm. **H** Box Plot of invaded area of WM266.4 spheroids treated with treated with DMSO, 3I, 3I plus recombinant PMEL amyloid fibrils (3I + PMEL), Verteporfin, Verteporfin plus 3I and Verteporfin plus 3I and recombinant PMEL amyloid fibrils at 48 h. *N* = 3 replicates. *T*-test analysis: *P < 0.05, **P < 0.01, ***P < 0.001. Data are presented as mean ± SD. **I** Representative model system of amyloid-driven YAP activation in cancer and its phenotypic effect.

YAP is an oncoprotein with different roles in cancer progression such as cell proliferation and stem cell maintenance [14]. In melanoma, it also affects the migration and invasion capacity [43] of cancer cells. Having demonstrated that PMEL amyloid fibrils increase YAP activation, we wondered if amyloid fibrils have an impact on migration and invasion capacity. Hence, metastatic melanoma cells (IGR37 and WM266.4) treated with BACE2 inhibitor showed reduction in migration capacity, which was rescued by the administration of rPMEL amyloid fibrils (Fig. S5A–D). To prove that the increased migration capacity exerted by amyloid fibrils depends on YAP activation, we performed a wound healing assay treating IGR37 cells with Verteporfin, a drug that displaces the interaction between YAP and TAZ thus impeding their transcriptional activity [44]. Indeed, when we administered Verteporfin, which per se decreases the migration of melanoma cells, both 3I and rPMEL are no longer able to modify the migration capacity of melanoma cells (Fig. 6A, B). Further, primitive melanoma cells treated with rPMEL amyloid fibrils migrate faster compared to untreated cells while the concomitant administration of Verteporfin abrogates the effect of rPMEL amyloid fibrils (Fig. 6C, D). In order to test if this effect is shared by pancreatic cancer cells, we analyzed BXPC3 behavior by wound healing assay. Consistently with data on melanoma, BACE2 inhibition reduces the migration capacity of pancreatic cancer cells, which is then rescued by the concomitant administration of Aβ40 peptide. The treatment with Verteporfin, also in BXPC3, abrogates the effects of amyloid fibrils on migration. (Fig. 6E, F). These results demonstrate that the influence of amyloid fibrils on cancer cells migration is shared by different cancer types, or at least by melanoma and pancreatic cancer cells and that YAP is necessary to drive this phenotype.

Finally, we also assessed whether amyloid fibrils could influence the invasive capacity of melanoma cells. To this aim, we established metastatic melanoma spheroids. We determined the presence of amyloid aggregates in this 3D culture by Proteostat staining and verified that amyloid aggregates are BACE2-dependent, as previously demonstrated in 2D cell cultures (Fig. S5E, F).

BACE2 inhibition decreases the ability of melanoma spheroids to invade the surrounding Matrigel, and this effect is reverted by embedding rPMEL amyloid fibrils into the Matrigel (Fig. S5G). To demonstrate that also the increased invasion capacity by amyloid fibrils is under the control of YAP, we administered Verteporfin to the melanoma spheroids culture and we observed a reduced invasion capacity of melanoma spheroids, while the amount of protein aggregates was not altered by the treatment (Fig. S5H, I). Moreover, both 3I and rPMEL fibrils administration are no more able to inhibit or promote spheroids invasion of the surrounding Matrigel (Fig. 6E, F). These results suggest that PMEL amyloid fibrils can modify the Matrigel, promoting the invasion of 3D melanoma spheroids in a YAP-dependent manner.

Finally, as mechanotransduction modulates also cancer cell proliferation and we previously observed that BACE2 inhibition affects melanoma cell proliferation [10], we wondered if amyloids-induced YAP activation might contribute also to tumor proliferation. Notably, we observed that BACE2 inhibition significantly reduced proliferation in both melanoma and PDAC cells, but the presence of amyloid fibrils did not counteract this inhibitory effect (Fig. S6A, B).

In addition, apoptosis rates remained unaffected by either BACE2 inhibition or amyloid fibrils (Fig S6C, D). Intriguingly, our investigation into cell cycle dynamics revealed that BACE2 inhibition led to alterations in cell cycle phases. In IGR37 melanoma cells, there was a G1 phase accumulation and a corresponding reduction of the S phase. However, the introduction of amyloid fibrils did not rescue this cell cycle phenotype (Fig. S6E). Similarly, in BXPC3 cells, BACE2 inhibition resulted in reduced S phase and an accumulation in G2, with amyloid fibrils failing to mitigate these observed effects (Fig. S6F).

These findings collectively underscore the non-toxic nature of BACE2 inhibition and suggest its role in regulating cell cycle progression. However, the contribution of BACE2 inhibition to the observed reduction in proliferation remains an intriguing aspect that warrants further in-depth investigation.

Overall, in this study, we discovered that in melanoma and pancreatic cancer, amyloid fibrils activate YAP nuclear translocation through a glycocalyx-HIPPO signaling axis, promoting migration and invasion of cancer cells (Fig. 6E). These results open the possibility to use inhibitors of amyloid maturation to dampen the detrimental effects of mechanotransduction in cancer.

## Discussion

Cancer-secreted soluble factors play several roles in the tumor microenvironment as they can have both autocrine and paracrine pro-tumorigenic effects [5]. Thus, interfering with secretion or modulating the effects of these factors has emerged as therapeutic priority in recent years. However, the identification of cancer-secreted soluble factors is challenging both in vivo and in vitro [45]. Recently, we developed a protocol for secretome analysis that allowed a comprehensive identification of melanoma-secreted proteins [10, 27]. Thanks to this method, we discovered that metastatic melanoma cells secrete amyloidogenic proteins and that the presence of amyloid aggregates in the secretome leads to YAP activation [10].

YAP is a mechanosensor with several pro-tumorigenic functions such as sustenance of proliferation, migration, invasion, stem cells maintenance, and dug resistance [14]. YAP inhibition may therefore represent a promising therapy to tackle cancer growth. Direct targeting of YAP, though, can have several side effects as YAP is involved in organ size definition and wound repair mechanisms [46, 47]. Hence, finding a way to inhibit YAP specifically in cancer cells can be highly efficacious and beneficial for cancer patients.

In this study, we report that amyloid accumulation occurs in different tumor biopsies. We also demonstrate that the amyloid fibril-dependent mechanoresponse, described for the first time in melanoma [10], is also conserved in PDAC. Till now, how amyloid fibrils activate mechanotransduction remained elusive.

In order to identify the main actors in amyloid-driven mechanotransduction, we searched for amyloid fibril interactors. Native immunoprecipitation of amyloid fibrils is not technically feasible due to their insolubility [48]. Hence, we took advantage of the insoluble nature of amyloid fibrils [49] and we purified the insoluble secretome of melanoma and PDAC cells. Analysis of the insoluble secretome identified Agrin as a bona fide amyloid fibril interactor. Agrin is a glycoprotein that belongs to the glycocalyx [34], a membrane bound layer of proteoglycans and glycoproteins that participate in mechanotransduction [36]. In particular, it was demonstrated that the glycocalyx perceives the interstitial flow shear, thus activating mechanotransduction pathways with consequences on cell migration, metastasis formation, cell adhesion, and tumor growth [50–52]. As Agrin was reported to drive mechanotransduction and to interact with Aβ peptides [41] becoming part of the amyloid plaque in Alzheimer’s disease brain [40], it seemed to be the perfect bridge between amyloids and mechanotransduction. Indeed, through functional assays we demonstrated that Agrin is the sensor of extracellular amyloid fibrils which act as a local signal that is able to induce mechanotransduction.

In hepatocellular carcinoma, it was reported that Agrin senses changes in ECM stiffness [39]. When the ECM stiffness increases, Agrin binds its receptors MuSK and LRP4, thus driving the assembly of focal adhesions that, through both ROCK-dependent cytoskeleton remodeling and HIPPO pathway deactivation, promote YAP nuclear translocation [39]. Instead, in our study, we demonstrated a unique signaling cascade that specifically relies on the deactivation of LATS1.

Finally, we also explored the phenotypic outcome of the deposition of amyloid fibrils, describing for the first time that amyloid fibrils are drivers of tumor cell migration and invasion in a YAP-dependent fashion.

In recent years, different research groups identified amyloids as part of the tumor microenvironment by studying its paracrine effects. Indeed, Aβ40 was shown to induce NETosis [23] or dampen neuroinflammation [24].

We are convinced that elucidation of the paracrine effects of amyloid-driven mechanoresponse on tumor microenvironment could further highlight the fundamental role played by amyloid fibrils in cancer progression.

Overall, in this study we describe amyloid fibrils as new components of the tumor microenvironment where they provide a mechanical stimulus, which results in the glycocalyx and HIPPO-dependent stimulation of YAP transcriptional activity affecting cancer cell migration and invasion. Whether this recognition is based on the intrinsic rigidity of amyloid fibrils or in a structural-based sensing is still to be clarified.

Taken together these findings suggest that inhibition of amyloid deposition, until now taken into consideration only for the treatment of Alzheimer’s disease [53], could represent a new strategy to counteract cancer development and progression.

### Supplementary information

Supplementary material

Table S1

Table S2

Table S3

Original data

## Data Availability

The mass spectrometry proteomics data have been deposited to the ProteomeXchange Consortium via the PRIDE [54] partner repository with the dataset identifier PXD041249.

## References

[1] Jin MZ, Jin WL (2020). The updated landscape of tumor microenvironment and drug repurposing. Signal Transduct Target Ther.

[2] Whiteside TL (2008). The tumor microenvironment and its role in promoting tumor growth. Oncogene.

[3] Labani-Motlagh A, Ashja-Mahdavi M, Loskog A (2020). The tumor microenvironment: a milieu hindering and obstructing antitumor immune responses. Front Immunol.

[4] Ansell SM, Vonderheide RH. Cellular composition of the tumor microenvironment. American Society of Clinical Oncology Educational Book. 2013;33:e91–e97. 10.14694/EdBook_AM.2013.33.e91 23714465

[5] Karagiannis GS, Pavlou MP, Diamandis EP (2010). Cancer secretomics reveal pathophysiological pathways in cancer molecular oncology. Mol Oncol.

[6] Galli F, Aguilera JV, Palermo B, Markovic SN, Nistico P, Signore A (2020). Relevance of immune cell and tumor microenvironment imaging in the new era of immunotherapy. J Exp Clin Cancer Res.

[7] Zhou C, Liu Q, Xiang Y, Gou X, Li W (2022). Role of the tumor immune microenvironment in tumor immunotherapy. Oncol Lett.

[8] Hirata E, Girotti MR, Viros A, Hooper S, Spencer-Dene B, Matsuda M (2015). Intravital imaging reveals how BRAF inhibition generates drug-tolerant microenvironments with high integrin beta1/FAK signaling. Cancer Cell.

[9] Sahai E, Astsaturov I, Cukierman E, DeNardo DG, Egeblad M, Evans RM (2020). A framework for advancing our understanding of cancer-associated fibroblasts. Nat Rev Cancer.

[10] Matafora V, Farris F, Restuccia U, Tamburri S, Martano G, Bernardelli C (2020). Amyloid aggregates accumulate in melanoma metastasis modulating YAP activity. EMBO Rep.

[11] Dupont S, Morsut L, Aragona M, Enzo E, Giulitti S, Cordenonsi M (2011). Role of YAP/TAZ in mechanotransduction. Nature.

[12] Walker C, Mojares E, Del Rio Hernandez A (2018). Role of extracellular matrix in development and cancer progression. Int J Mol Sci.

[13] Dupont S (2016). Role of YAP/TAZ in cell-matrix adhesion-mediated signalling and mechanotransduction. Exp Cell Res.

[14] Zanconato F, Cordenonsi M, Piccolo S (2016). YAP/TAZ at the roots of cancer. Cancer Cell.

[15] Piccolo S, Dupont S, Cordenonsi M (2014). The biology of YAP/TAZ: hippo signaling and beyond. Physiol Rev.

[16] Liu-Chittenden Y, Huang B, Shim JS, Chen Q, Lee SJ, Anders RA (2012). Genetic and pharmacological disruption of the TEAD-YAP complex suppresses the oncogenic activity of YAP. Genes Dev.

[17] Zhang Z, Lin Z, Zhou Z, Shen HC, Yan SF, Mayweg AV (2014). Structure-based design and synthesis of potent cyclic peptides inhibiting the YAP-TEAD protein-protein interaction. ACS Med Chem Lett.

[18] Song S, Xie M, Scott AW, Jin J, Ma L, Dong X (2018). A novel YAP1 inhibitor targets CSC-enriched radiation-resistant cells and exerts strong antitumor activity in esophageal adenocarcinoma. Mol Cancer Ther.

[19] Fitzpatrick AW, Park ST, Zewail AH (2013). Exceptional rigidity and biomechanics of amyloid revealed by 4D electron microscopy. Proc Natl Acad Sci USA.

[20] Zayas‐Santiago A, Martínez‐Montemayor MM, Colón‐Vázquez J, Ortiz‐Soto G, Cirino‐Simonet JG, Inyushin M (2022). Accumulation of amyloid beta (Aβ) and amyloid precursor protein (APP) in tumors formed by a mouse xenograft model of inflammatory breast cancer. FEBS Open Bio.

[21] van der Willik KD, Ghanbari M, Fani L, Compter A, Ruiter R, Stricker BHC (2020). Higher plasma amyloid-β levels are associated with a higher risk of cancer: a population-based prospective cohort study the association between amyloid-β and cancer. Cancer Epidemiol Biomark Prev.

[22] Li J, Guo M, Chen L, Chen Z, Fu Y, Chen Y (2022). p53 amyloid aggregation in cancer: function, mechanism, and therapy. Exp Hematol Oncol.

[23] Munir H, Jones JO, Janowitz T, Hoffmann M, Euler M, Martins CP (2021). Stromal-driven and amyloid β-dependent induction of neutrophil extracellular traps modulates tumor growth. Nat Commun.

[24] Kleffman K, Levinson G, Rose IV, Blumenberg LM, Shadaloey SA, Dhabaria A (2022). Melanoma-secreted amyloid beta suppresses neuroinflammation and promotes brain metastasis. Cancer Discov.

[25] Ronchi A, Marino FZ, Toni G, Pagliuca F, Russo D, Signoriello G (2022). Diagnostic performance of melanocytic markers for immunocytochemical evaluation of lymph-node melanoma metastases on cytological samples. J Clin Pathol.

[26] Zhang S, Chen K, Liu H, Jing C, Zhang X, Qu C (2021). PMEL as a prognostic biomarker and negatively associated with immune infiltration in skin cutaneous melanoma (SKCM). J Immunother.

[27] Matafora V, Bachi A (2020). Secret3D workflow for secretome analysis. STAR Protoc.

[28] Farris F, Matafora V, Bachi A (2021). The emerging role of beta-secretases in cancer. J Exp Clin Cancer Res.

[29] Farzan M, Schnitzler CE, Vasilieva N, Leung D, Choe H (2000). BACE2, a β-secretase homolog, cleaves at the β site and within the amyloid-β region of the amyloid-β precursor protein. Proc Natl Acad Sci USA.

[30] Stutzer I, Selevsek N, Esterhazy D, Schmidt A, Aebersold R, Stoffel M (2013). Systematic proteomic analysis identifies beta-site amyloid precursor protein cleaving enzyme 2 and 1 (BACE2 and BACE1) substrates in pancreatic beta-cells. J Biol Chem.

[31] Hales CM, Dammer EB, Deng Q, Duong DM, Gearing M, Troncoso JC (2016). Changes in the detergent-insoluble brain proteome linked to amyloid and tau in Alzheimer’s disease progression. Proteomics.

[32] Kayed R, Head E, Sarsoza F, Saing T, Cotman CW, Necula M (2007). Fibril specific, conformation dependent antibodies recognize a generic epitope common to amyloid fibrils and fibrillar oligomers that is absent in prefibrillar oligomers. Mol Neurodegener.

[33] Varadi M, De Baets G, Vranken WF, Tompa P, Pancsa R (2018). AmyPro: a database of proteins with validated amyloidogenic regions. Nucleic Acids Res.

[34] Kang H, Wu Q, Sun A, Liu X, Fan Y, Deng X (2018). Cancer cell glycocalyx and its significance in cancer progression. Int J Mol Sci.

[35] Huang ML, Tota EM, Lucas TM, Godula K (2018). Influencing early stages of neuromuscular junction formation through glycocalyx engineering. ACS Chem Neurosci.

[36] Iozzo RV, Sanderson RD (2011). Proteoglycans in cancer biology, tumour microenvironment and angiogenesis. J Cell Mol Med.

[37] Tarbell JM, Pahakis M (2006). Mechanotransduction and the glycocalyx. J Intern Med.

[38] Pahakis MY, Kosky JR, Dull RO, Tarbell JM (2007). The role of endothelial glycocalyx components in mechanotransduction of fluid shear stress. Biochem Biophys Res Commun.

[39] Chakraborty S, Njah K, Pobbati AV, Lim YB, Raju A, Lakshmanan M (2017). Agrin as a mechanotransduction signal regulating YAP through the Hippo pathway. Cell Rep.

[40] Verbeek MM, Otte-Höller I, van den Born J, van den Heuvel LP, David G, Wesseling P (1999). Agrin is a major heparan sulfate proteoglycan accumulating in Alzheimer’s disease brain. Am J Pathol.

[41] Cotman SL, Halfter W, Cole GJ (2000). Agrin binds to beta-amyloid (Abeta), accelerates abeta fibril formation, and is localized to Abeta deposits in Alzheimer’s disease brain. Mol Cell Neurosci.

[42] van Niel G, Bergam P, Di Cicco A, Hurbain I, Lo Cicero A, Dingli F (2015). Apolipoprotein E regulates amyloid formation within endosomes of pigment cells. Cell Rep.

[43] Zhang X, Yang L, Szeto P, Abali GK, Zhang Y, Kulkarni A (2020). The Hippo pathway oncoprotein YAP promotes melanoma cell invasion and spontaneous metastasis. Oncogene.

[44] Feng J, Gou J, Jia J, Yi T, Cui T, Li Z (2016). Verteporfin, a suppressor of YAP-TEAD complex, presents promising antitumor properties on ovarian cancer. Onco Targets Ther.

[45] Schira-Heinen J, Grube L, Waldera-Lupa DM, Baberg F, Langini M, Etemad-Parishanzadeh O (2019). Pitfalls and opportunities in the characterization of unconventionally secreted proteins by secretome analysis. Biochimica Biophys Acta Proteins Proteom.

[46] Cahill MT, Smith BT, Fekrat S (2002). Adverse reaction characterized by chest pain, shortness of breath, and syncope associated with verteporfin (visudyne). Am J Ophthalmol.

[47] Dey A, Varelas X, Guan K-L (2020). Targeting the Hippo pathway in cancer, fibrosis, wound healing and regenerative medicine. Nat Rev Drug Discov.

[48] Bonifacino JS, Gershlick DC, Dell’Angelica EC. Immunoprecipitation. Curr Protoc Cell Biol. 2016;71. 10.1002/cpcb.3 27245424

[49] Rostagno A, Ghiso J. Isolation and biochemical characterization of amyloid plaques and paired helical filaments. Current protocols in cell biology. 2009;44:3–33. 10.1002/0471143030.cb0333s44 PMC2793596 19731227

[50] Qazi H, Shi ZD, Song JW, Cancel LM, Huang P, Zeng Y (2016). Heparan sulfate proteoglycans mediate renal carcinoma metastasis. Int J Cancer.

[51] Shi ZD, Wang H, Tarbell JM (2011). Heparan sulfate proteoglycans mediate interstitial flow mechanotransduction regulating MMP-13 expression and cell motility via FAK-ERK in 3D collagen. PLoS ONE.

[52] Qazi H, Palomino R, Shi Z-D, Munn LL, Tarbell JM (2013). Cancer cell glycocalyx mediates mechanotransduction and flow-regulated invasion. Integr Biol.

[53] Wessels AM, Lines C, Stern RA, Kost J, Voss T, Mozley LH (2020). Cognitive outcomes in trials of two BACE inhibitors in Alzheimer’s disease. Alzheimer’s Dement.

[54] Perez-Riverol Y, Bai J, Bandla C, García-Seisdedos D, Hewapathirana S, Kamatchinathan S (2022). The PRIDE database resources in 2022: a hub for mass spectrometry-based proteomics evidences. Nucleic Acids Res.

