# Supplementary material for: Unveiling the mechanistic link between extracellular amyloid fibrils, mechano-signaling and YAP activation in cancer
Source: Cell Death Dis. 2024 Jan 11;15(1):28. doi: 10.1038/s41419-024-06424-z (PMC10781709; doi:10.1038/s41419-024-06424-z)
Supplement: Supplementary file 1 — Supplementary material [file 41419_2024_6424_MOESM1_ESM.docx]

Supplementary materials for

**Unveiling the mechanistic link between extracellular amyloid fibrils, mechano-signaling and YAP activation in cancer**

Francesco Farris^1^, Alice Elhagh^1^, Ilaria Vigorito^1^, Nicoletta Alongi^1,2^, Federica Pisati^3^, Michele Giannattasio^1,4^, Francesca Casagrande^1,5^, Lisa Veghini^6^, Vincenzo Corbo^6,7^, Claudio Tripodo^1,8^, Arianna Di Napoli^9^, Vittoria Matafora^1*^ and Angela Bachi^1*^

1 IFOM ETS – The AIRC Institute of Molecular Oncology, 20139 Milan, Italy

2 Present address: IRCCS Humanitas Research Hospital, Rozzano, Milan, Italy

3 Histopathology Unit, Cogentech S.C.a.R.L., 20139 Milan, Italy

4 Department of Oncology and Hemato-Oncology, University of Milan, 20122 Milan, Italy

5 Present address: Human Technopole, Milan, Italy

6 Department of Engineering for Innovation Medicine (DIMI), University of Verona, 37134, Verona, Italy

7 ARC-Net Centre for Applied Research on Cancer, University of Verona, 37134, Verona, Italy.

8 Tumor Immunology Unit, Department of Health Sciences, University of Palermo, 90133

Palermo, Italy

9 Pathology Unit, Department of Clinical and Molecular Medicine, Sant'Andrea University Hospital, Sapienza University of Rome, 00189 Rome, Italy.

* Corresponding author:

[angela.bachi@ifom.eu](mailto:angela.bachi@ifom.eu), vittoria.matafora@ifom.eu

Phone: +3902574303873

Figure S1.


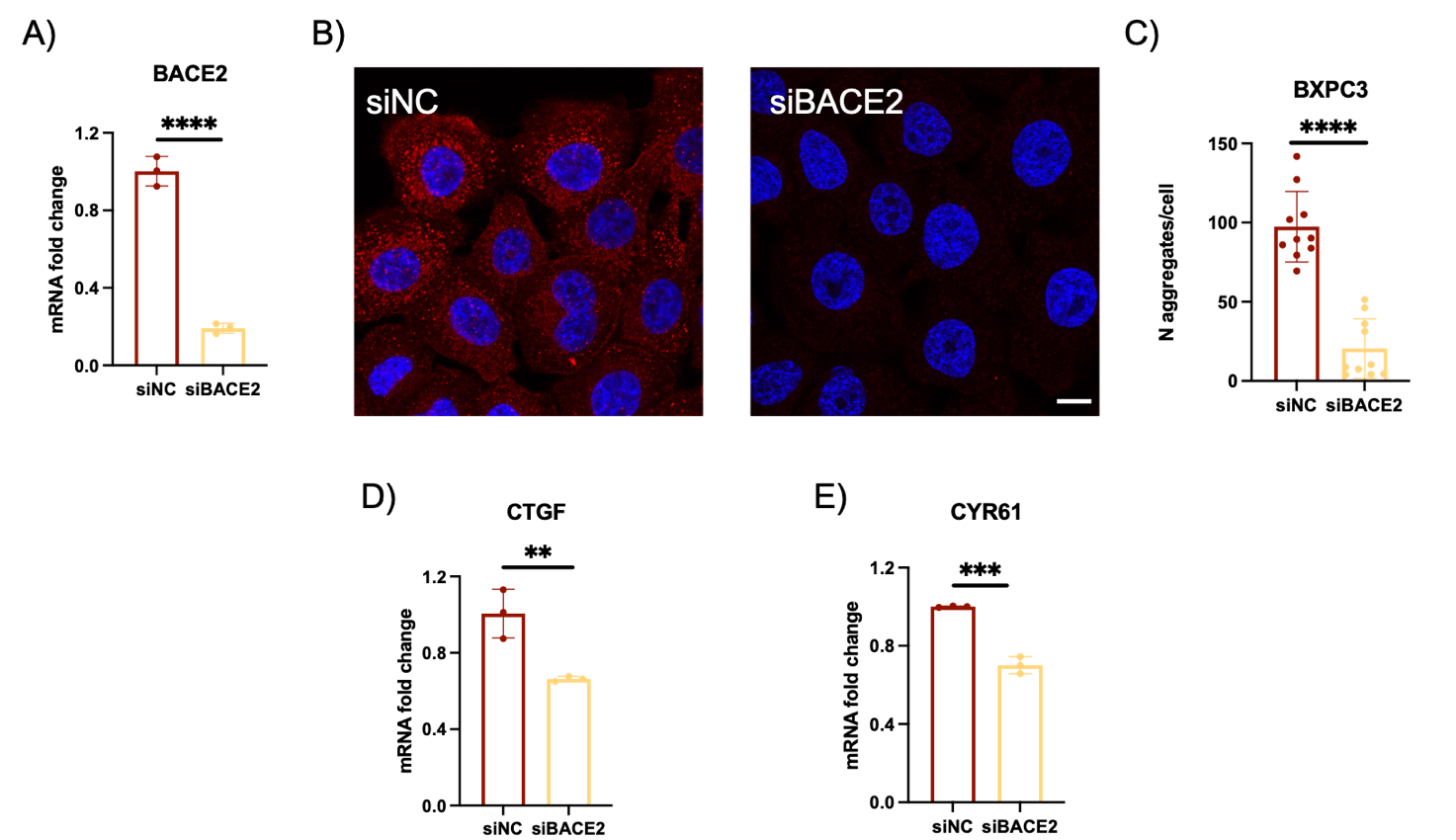


**Figure S1. BACE2 silencing in BXPC3 PDAC cell line**

(A) BACE2 mRNA fold change in BXPC3 cells transfected with siRNA against BACE2 (siBACE2) or siRNA non-coding (siNC). N = 3 biological replicates. T-test analysis: *****P* < 0.0001.

(B) Confocal fluorescence images of Proteostat (1:1,000, red) and DAPI staining (blue) in BXPC3 cells transfected with siRNA against BACE2 (siBACE2) or siRNA non-coding (siNC). Scale Bar 10 μm

(C) Quantification of amyloid aggregates detected by immunofluorescence in BXPC3 cells transfected with siRNA against BACE2 (siBACE2) or siRNA non-coding (siNC). The analysis is performed by Fiji software. N = 10. T-test, ****P < 0.0001. Data are presented as mean ± SD.

(D) CTGF mRNA fold change in BXPC3 WT (siNC) or BACE2 KD cells (siBACE2). N = 3 biological replicates. T-test analysis: ***P* < 0.01. Data are presented as mean ± SD.

(E) CYR61 mRNA fold change in BXPC3 WT (siNC) or BACE2 KD cells (siBACE2) treated or not with rPMEL amyloid fibrils. N = 3 biological replicates. T-test analysis: ****P* < 0.001. Data are presented as mean ± SD.

Figure S2.

**
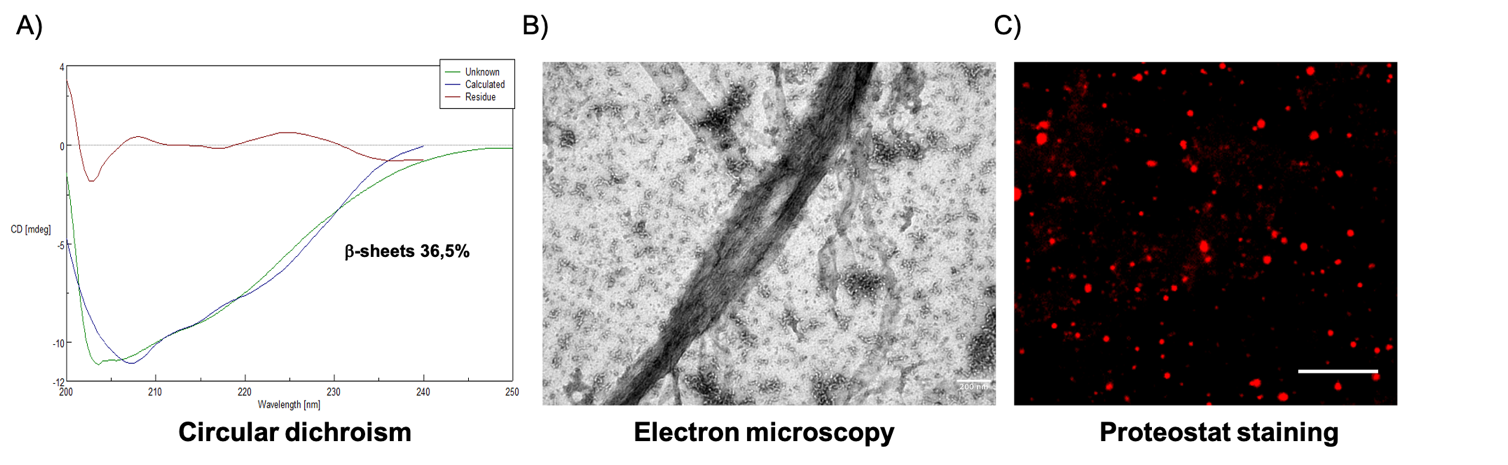
**

**Figure S2. rPMEL amyloid fibrils show common physical and chemical characteristics of amyloid fibrils.**

(A) Recombinant PMEL amyloid fibrils show a 36.5% $\beta$-sheets composition as measured by Circular dichroism.

(B) Electron microscopy micrograph of recombinant PMEL amyloid fibrils showing the twisted amyloid fibril structure of the protein. Scale bar: 200nm.

(C) Proteostat staining of recombinant PMEL amyloid fibrils, showing high positivity for the amyloidophilic dye. Scale bar: 10µm.

Figure S3.

**
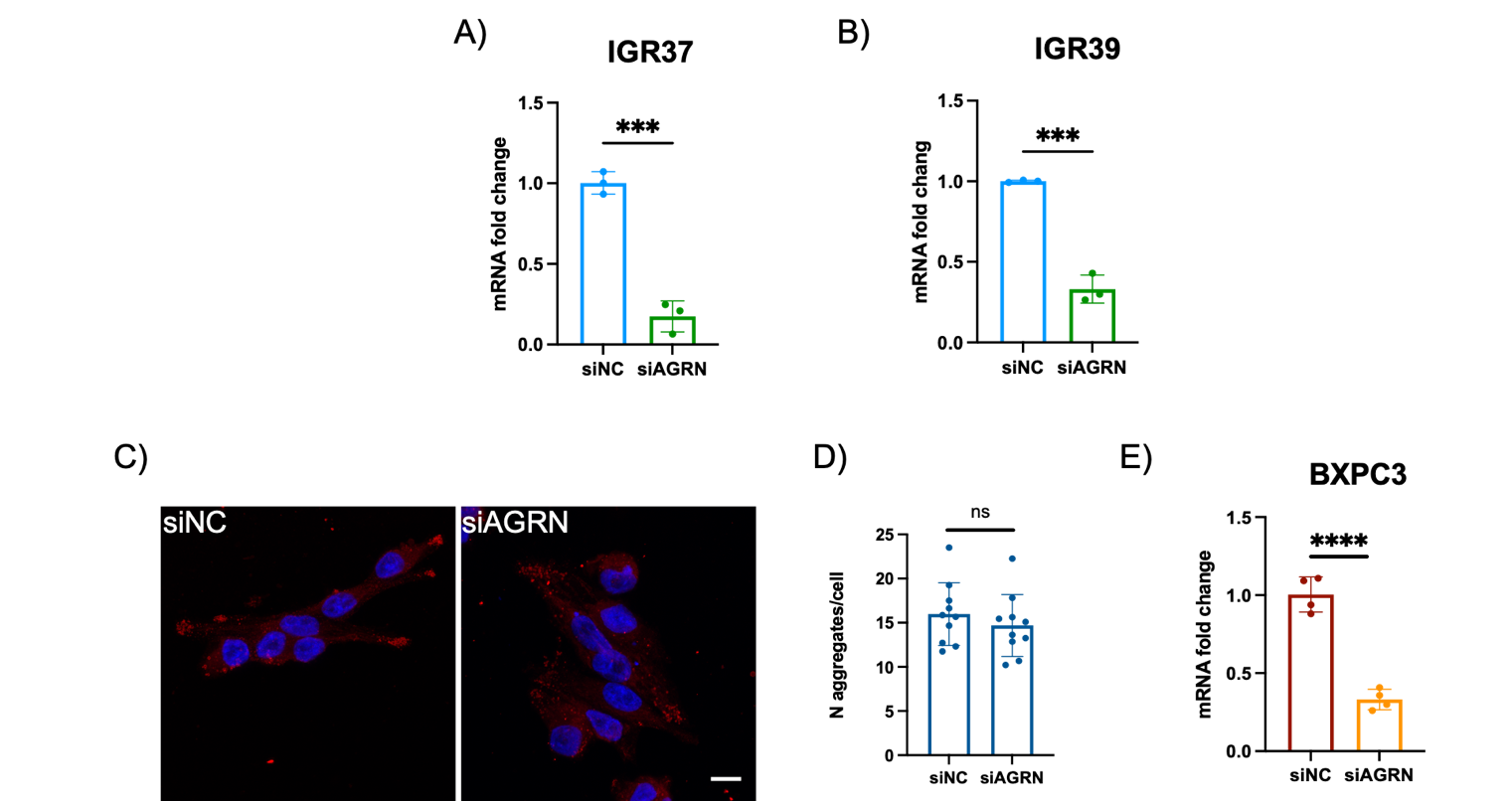
**

**Figure S3. Agrin silencing in melanoma and PDAC cells**

(A) AGRN mRNA fold change in IGR37 cells transfected with siRNA against Agrin (siAGRN) or siRNA non-coding (siNC). N = 3 biological replicates. T-test analysis: ****P* < 0.001.

(B) AGRN mRNA fold change in IGR39 cells transfected with siRNA against Agrin (siAGRN) or siRNA non-coding (siNC). N = 3 biological replicates. T-test analysis: ****P* < 0.001.

(C) Confocal fluorescence images of Proteostat (1:1,000, red) and DAPI staining (blue) in WT (CTRL) or Agrin KD (siAGRN) IGR37 cells. Scale Bar 10 μm

(D) Quantification of amyloid aggregates in WT (CTRL) or Agrin KD (siAGRN) IGR37 cells by immunofluorescence analysis using Fiji software. N = 10 biological replicates. T-test, ns > 0.05. Data are presented as mean ± SD.

(E) AGRN mRNA fold change in BXPC3 cells transfected with siRNA against Agrin (siAGRN) or siRNA non-coding (siNC). N = 3 biological replicates. T-test analysis: ****P* < 0.001.

**Figure S4.**

**
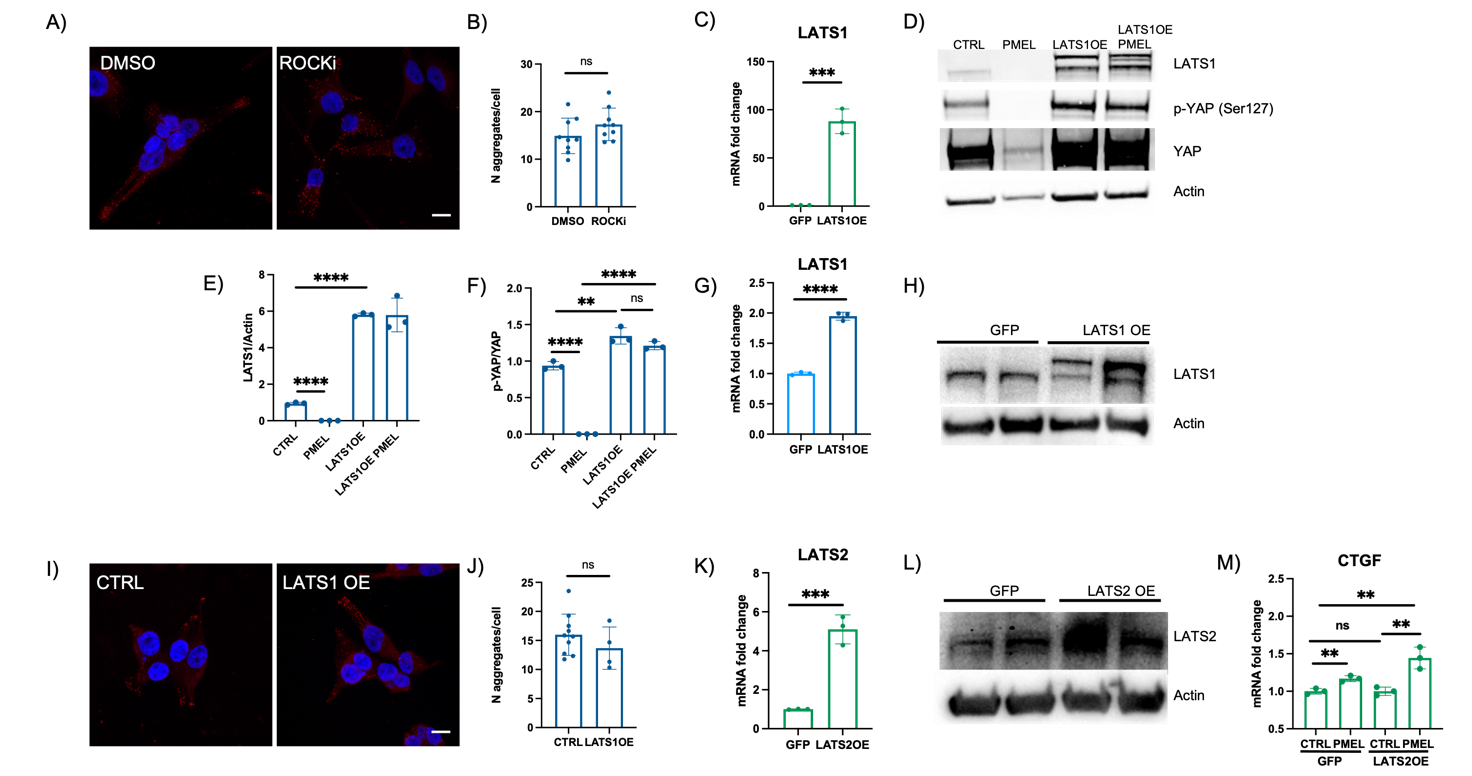
**

**Figure S4. Agrin silencing and LATS1/2 overexpression in melanoma and pancreatic cancer cells.**

(A) Confocal fluorescence images of Proteostat (1:1,000, red) and DAPI staining (blue) of IGR37 cells treated with DMSO or ROCK inhibitor (ROCKi). Scale Bar 10 μm

(B) Quantification of amyloid aggregates in IGR37 cells treated with DMSO or ROCK inhibitor (ROCKi) by immunofluorescence analysis using Fiji software. N = 9 biological replicates. T-test, ns > 0.05. Data are presented as mean ± SD.

(C) LATS1 mRNA fold change in IGR39 cells transfected with GFP or GFP-LATS1 overexpressing plasmid (LATS1OE).

(D) Western Blot of LATS1, p-YAP and YAP in IGR39 control (CTRL) cells, treated with rPMEL amyloid fibrils (PMEL), overexpressing LATS1 (LATS1OE) and overexpressing LATS1 treated with rPMEL amyloid fibrils (LATS1OE PMEL); actin was used as protein loading control.

(E) Quantification of LATS1 normalized on actin in IGR39 control (CTRL) cells, treated with rPMEL amyloid fibrils (PMEL), overexpressing LATS1 (LATS1OE) and overexpressing LATS1 treated with rPMEL amyloid fibrils (LATS1OE PMEL). N = 3 biological replicates. T-test, ***P* < 0.01. ****P* < 0.001. *****P* < 0.0001. Data are presented as mean ± SD.

(F) Quantification of pYAP normalized on YAP in IGR39 control (CTRL) cells, treated with rPMEL amyloid fibrils (PMEL), overexpressing LATS1 (LATS1OE) and overexpressing LATS1 treated with rPMEL amyloid fibrils (LATS1OE PMEL). N = 3 biological replicates. T-test, *****P* < 0.0001. Data are presented as mean ± SD.

(G) LATS1 mRNA fold change in IGR37 cells transfected with GFP or GFP-LATS1 overexpressing plasmid (LATS1OE).

(H) Western blot of LATS1 in IGR37 cells transfected with GFP or GFP-LATS1 overexpressing plasmid (LATS1OE). Actin was used as protein loading control.

(I) Confocal fluorescence images of Proteostat (1:1,000, red) and DAPI staining (blue) of IGR37 WT (CTRL) cells or LATS1 overexpressing cells (LATS1OE). Scale Bar 10 μm

(J) Quantification of amyloid aggregates in IGR37 WT (CTRL) or LATS1 overexpressing cells (LATS1OE) by immunofluorescence analysis using Fiji software. N = 4 biological replicates. T-test, ns > 0.05. Data are presented as mean ± SD.

(K) LATS2 mRNA fold change in IGR39 cells transfected with GFP or LATS2 overexpressing plasmid (LATS2OE).

(L) Western blot of LATS2 in IGR39 cells transfected with GFP or LATS2 overexpressing plasmid (LATS2OE). Actin was used as protein loading control.

(M) CTGF mRNA level in IGR39 WT (GFP) or LATS2 overexpressing cells (LATS2OE) treated or not with rPMEL amyloid fibrils. ^ns^ *P* > 0.05, ***P* < 0.01. Data are presented as mean ± SD.

Figure S5.

**
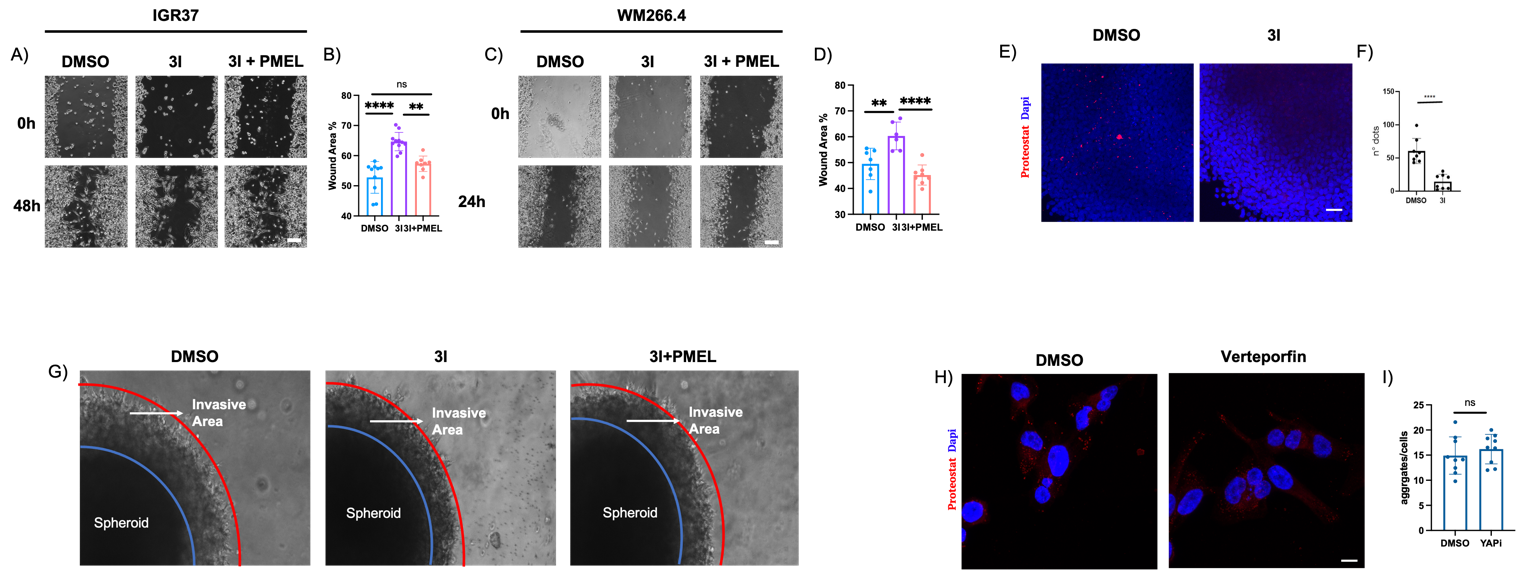
**

**Figure S5. Migration and invasion assay of melanoma treated with 3I and PMEL amyloid fibrils**

(A) Representative micrographs of IGR37 cells treated with DMSO or 3I, or 3I and recombinant PMEL amyloid fibrils (3I+PMEL) in a wound healing assay at 0 and 48 hours. Scale Bar 100 μm

(B) Box Plot of wound area for IGR37 cells treated with DMSO or 3I, or 3I and recombinant PMEL amyloid fibrils (3I+PMEL) at different time points. N = 10 replicates. T-test analysis: ***P* < 0.01, *****P* < 0.0001. Data are presented as mean ± SD.

(C) Representative micrographs of WM226.4 cells treated with DMSO or 3I, or 3I and recombinant PMEL amyloid fibrils (3I+PMEL) in a wound healing assay at 0 and 24 hours. Scale Bar 100 μm

(D) Box Plot of wound area for WM266.4 cells treated with DMSO or 3I, or 3I and recombinant PMEL amyloid fibrils (3I+PMEL) at different time points. N = 7 replicates. T-test analysis: ***P* < 0.01, *****P* < 0.0001. Data are presented as mean ± SD.

(E) Confocal fluorescence images of Proteostat (1:1,000, red) and DAPI staining (blue) of spheroids derived from WM266.4 and treated with DMSO or 3I.

(F) Quantification of amyloid aggregates in metastatic melanoma spheroids treated or not with 3I for 48 hours by immunofluorescence analysis using Fiji software. N = 8 replicates. T-test, *****P* < 0.0001. Data are presented as mean ± SD.

(G) Magnification of the invasive area of spheroids treated with DMSO or 3I for 48 hours, or 3I and recombinant PMEL amyloid fibrils (3I+PMEL) for 48 hours.

(H) Confocal fluorescence images of Proteostat (1:1,000, red) and DAPI staining (blue) of IGR37 cells treated with DMSO or Verteporfin. Scale Bar 10 μm

(I) Quantification of amyloid aggregates in IGR37 cells treated with DMSO or Verteporfin by immunofluorescence analysis using Fiji software. N = 9 biological replicates. T-test, ns > 0.05. Data are presented as mean ± SD.

**Figure S6.**

**
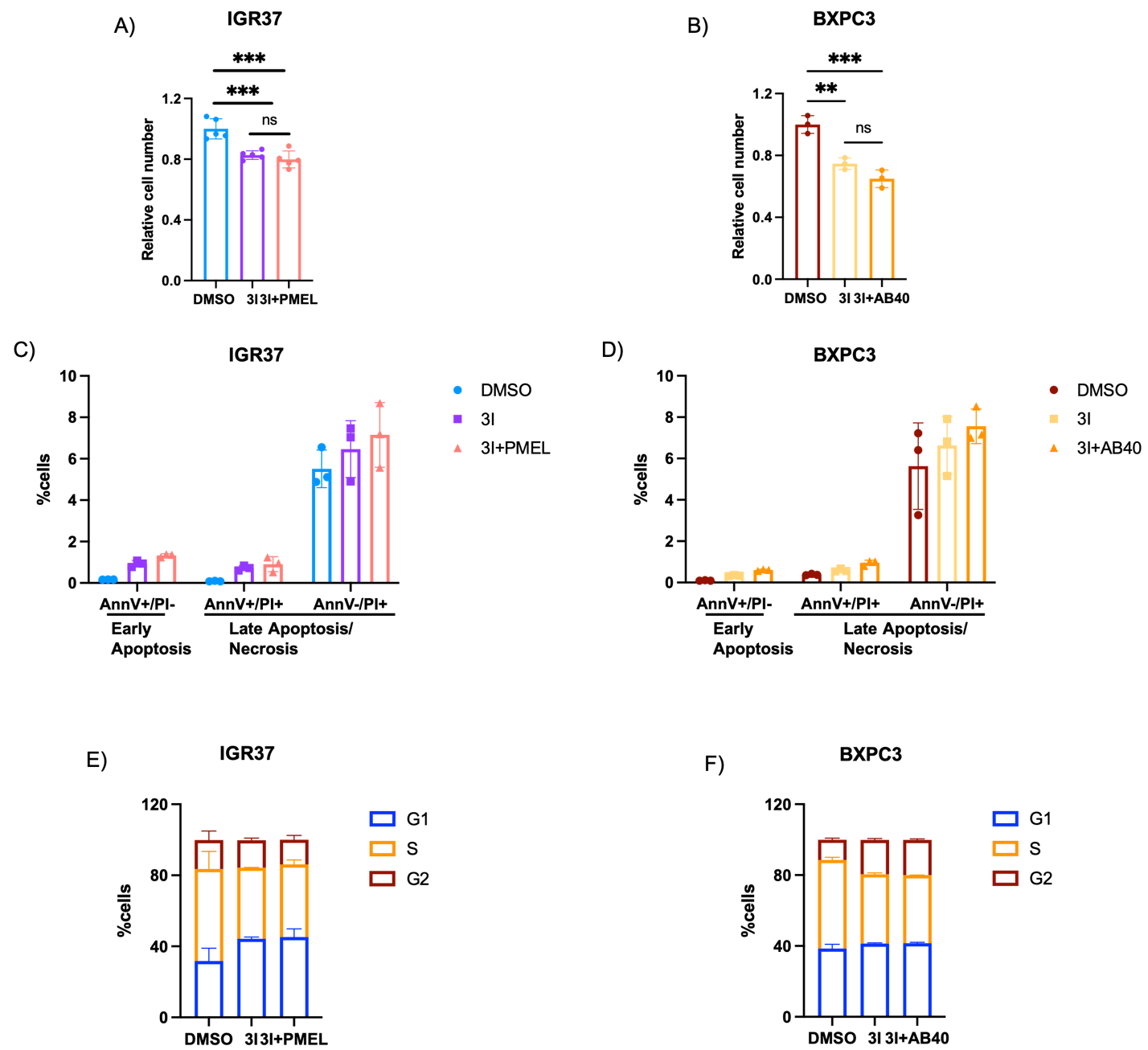
**

**Figure S6. Cell number, apoptosis e cell cycle analysis of melanoma and PDAC cells treated with 3I and PMEL amyloid fibrils**

(A) Cell Viability of IGR37 cells treated with 3I alone or in combination with rPMEL amyloid fibrils. N= 5 biological replicates. T-test, ns P>0,05. ****P* < 0.001. Data are presented as mean ± SD.

(B) Cell Viability of BXPC3 cells treated with 3I alone or in combination with Aβ40. N= 3 biological replicates. T-test, ns P>0,05. ***P* < 0.01. ****P* < 0.001. Data are presented as mean ± SD.

(C) Flow cytometry analysis of early apoptosis (AnnV+, PI-) and late apoptosis/necrosis (AnnV+, PI+; AnnV-, PI+) in IGR37 cells treated with 3I alone or in combination with rPMEL amyloid fibrils. N=3 biological replicates. Data are presented as mean ± SD.

(D) Flow cytometry analysis of early apoptosis (AnnV+, PI-) and late apoptosis/necrosis (AnnV+, PI+; AnnV-, PI+) in BXPC3 treated with 3I alone or in combination with Aβ40. N=3 biological replicates. Data are presented as mean ± SD.

(E) Flow cytometry analysis of cell cycle in IGR37 cells treated with 3I alone or in combination with rPMEL amyloid fibrils. N=3 biological replicates. Data are presented as mean ± SD.

(F) Flow cytometry analysis of cell cycle in BXPC3 cells treated with 3I alone or in combination with Aβ40. N=3 biological replicates. Data are presented as mean ± SD.

Table S1. Secretome proteomics of BXPC3 treated with DMSO versus 3I; List of significant protein (p-value=0,05) in BXPC3 cell line secretome tyreated with DMSO or 3I.

Table S2. Insoluble fraction secretomics of IGR37 and BXPC3; Proteins identified in insoluble secretome of IGR37 and BXPC3 cell line.

Table S3. Interactomics of recombinant PMEL amyloid fibrils; Proteins statistically significant in PMEL pull-down versus CTRL.
