# Supplementary material for: Unveiling the mechanistic link between extracellular amyloid fibrils, mechano-signaling and YAP activation in cancer
Source: Cell Death Dis. 2024 Jan 11;15(1):28. doi: 10.1038/s41419-024-06424-z (PMC10781709; doi:10.1038/s41419-024-06424-z)

Source Fig.2B

BXPC3-ASPC1

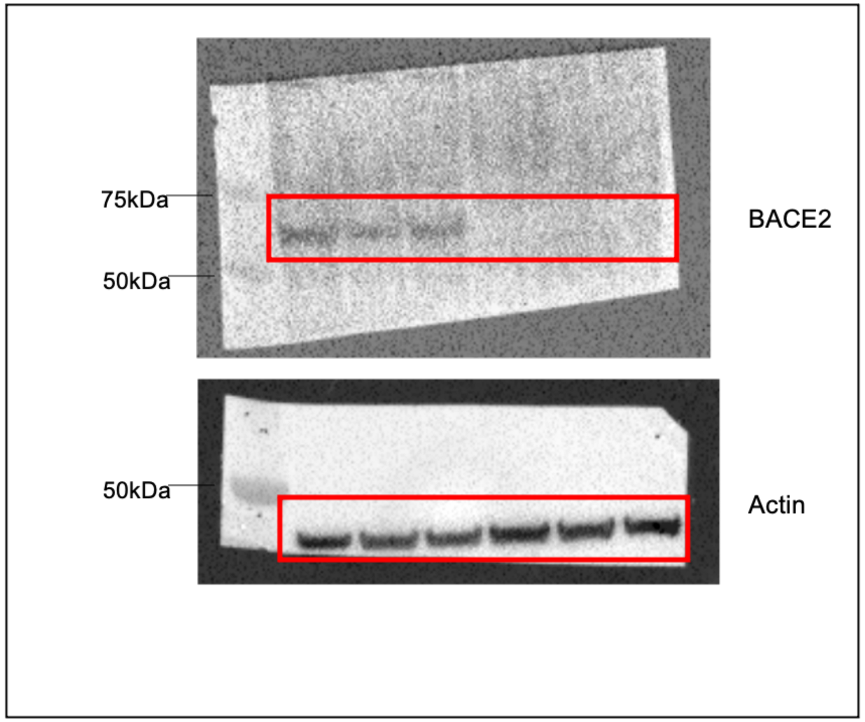

Capan1-Capan2

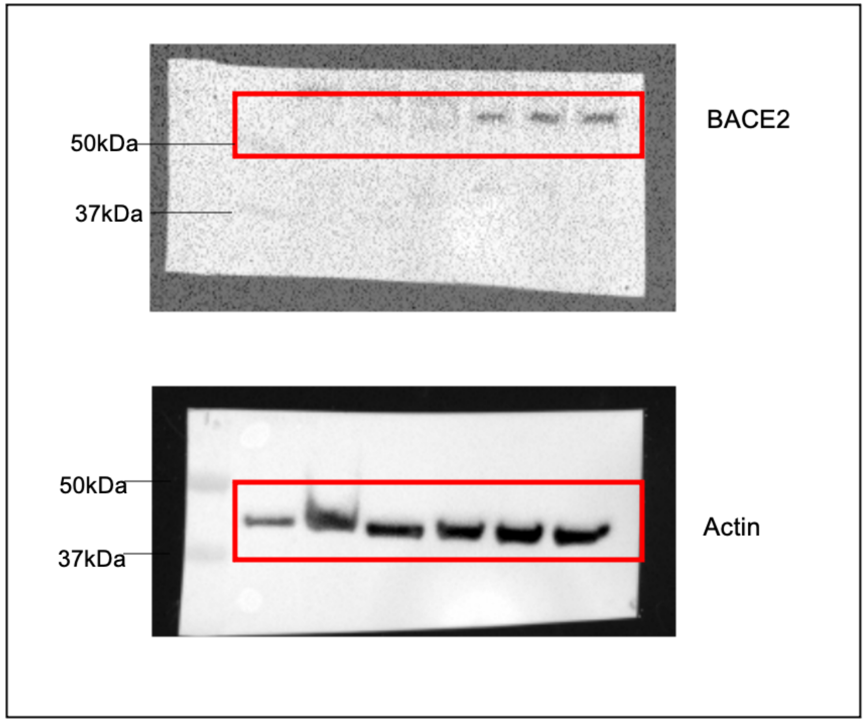

PANC1-Su86.86

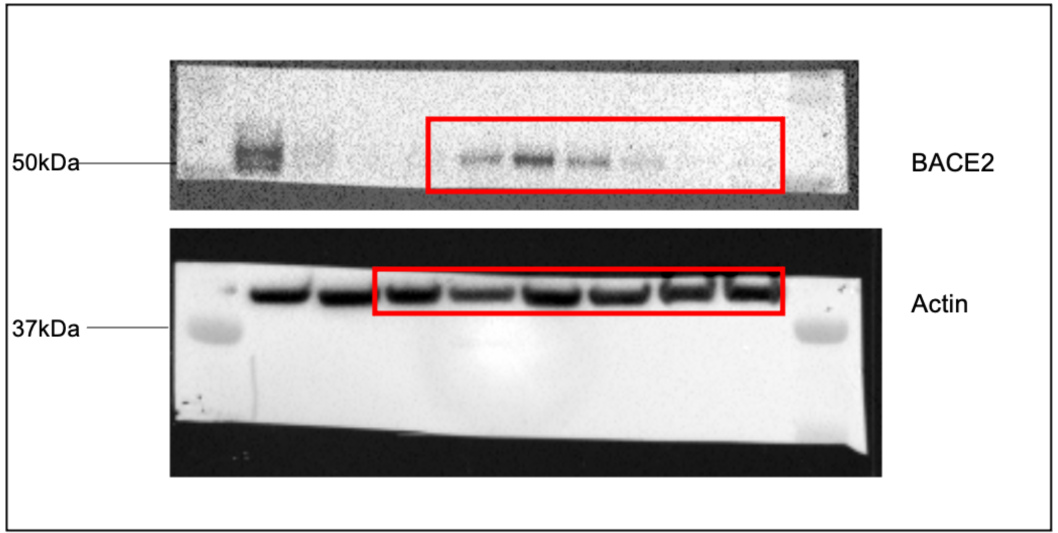

Source
Fig.3

Fig. 3A

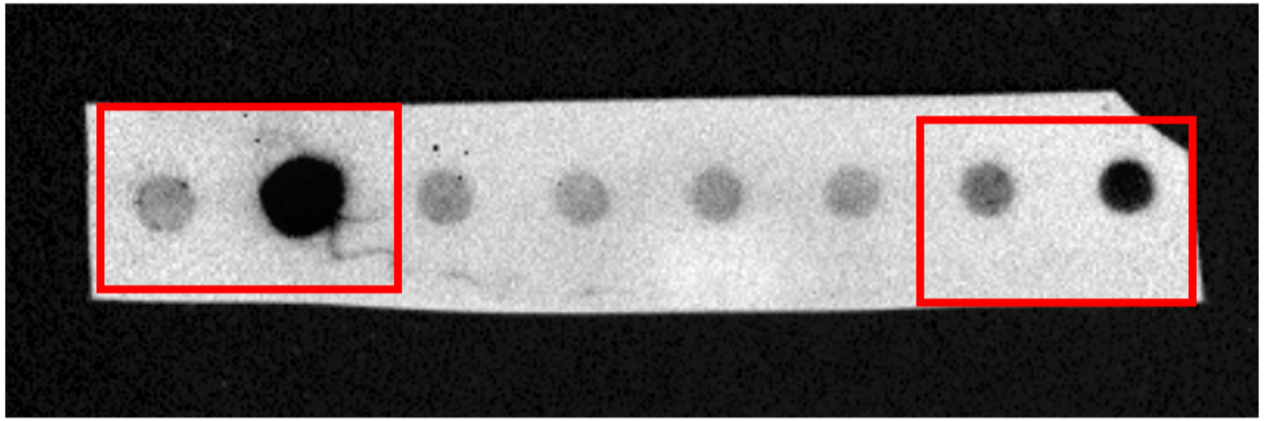

Fig. 3C

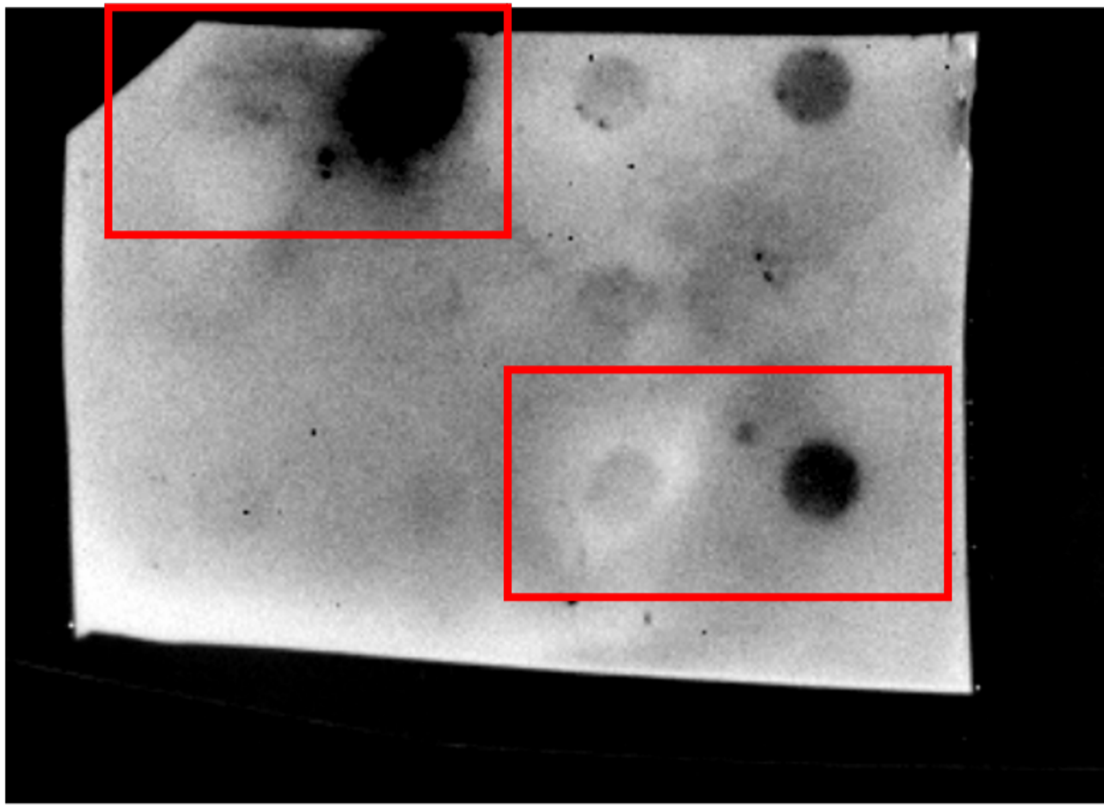

IB: OC

Source WB Fig. S4D

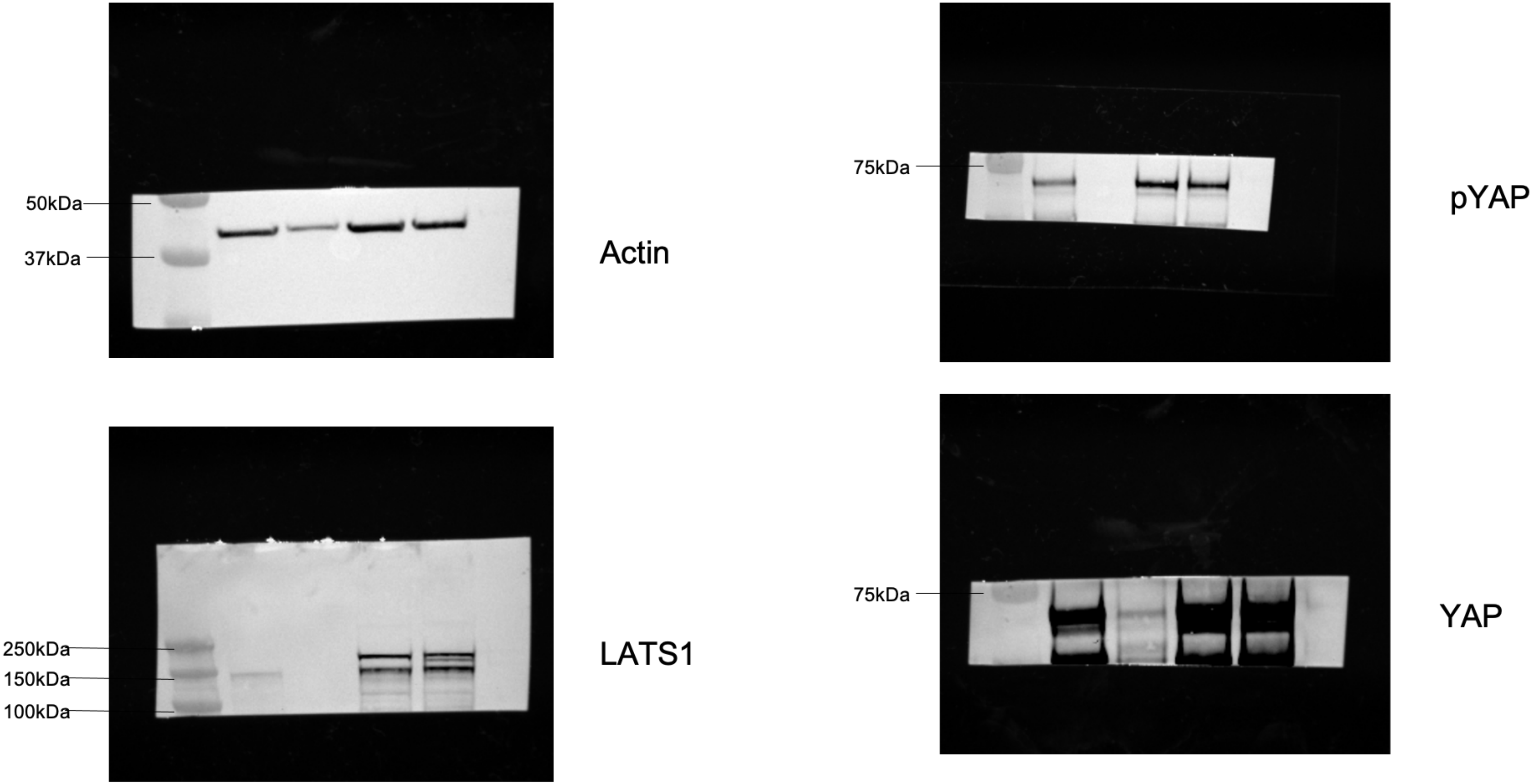

Source Fig. S4

H)

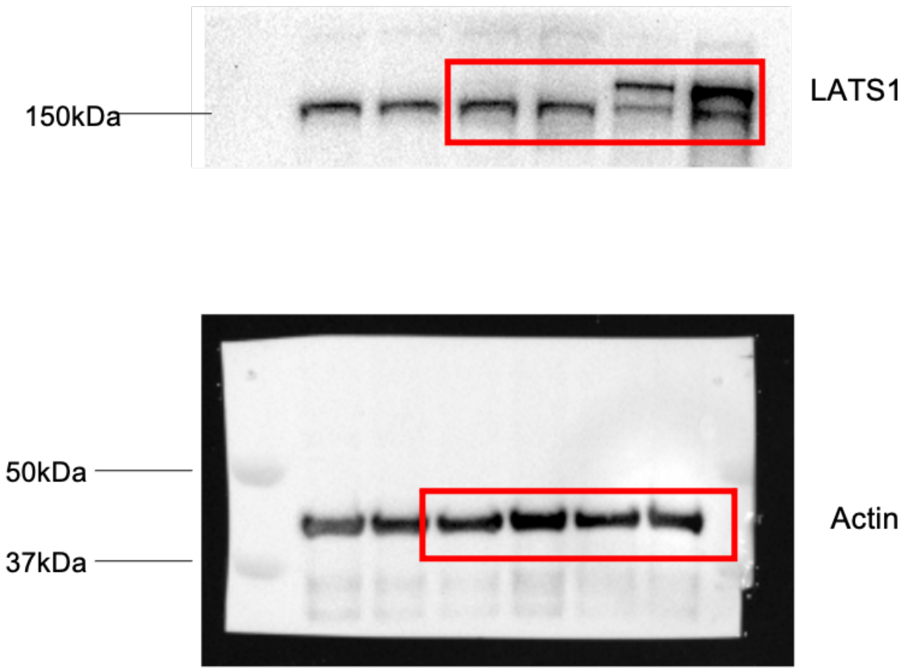

L)

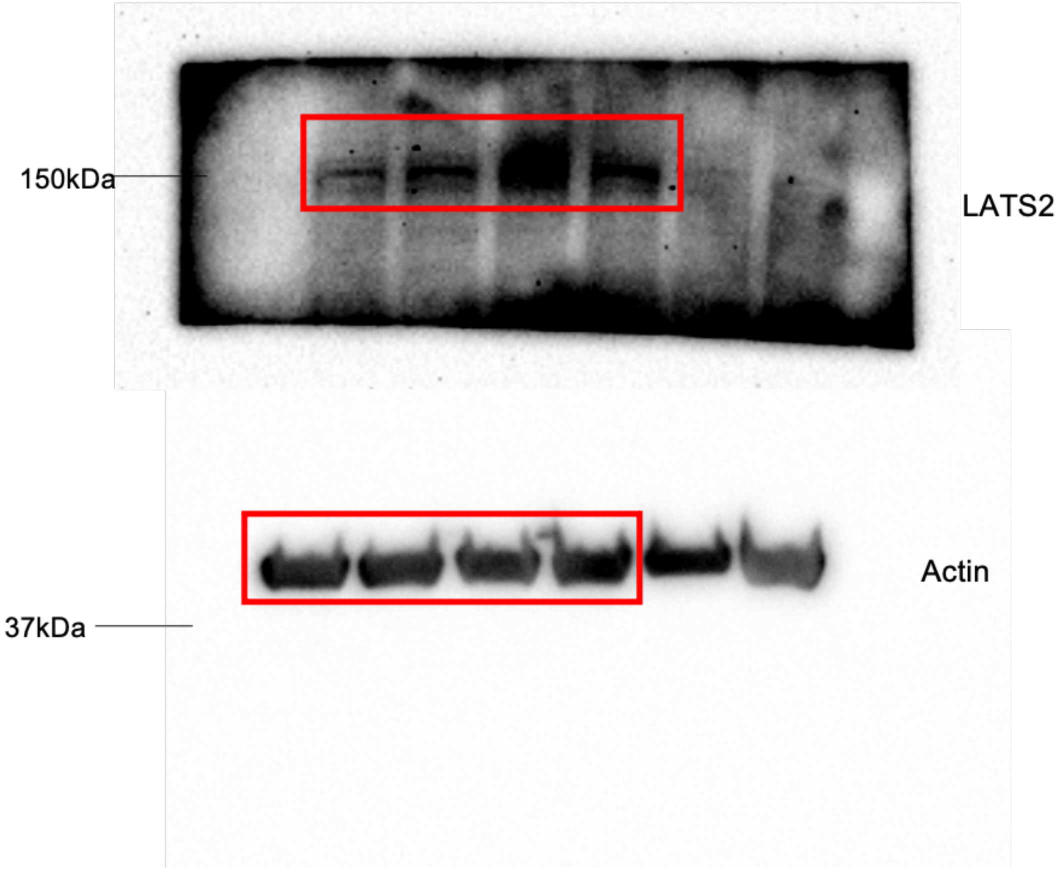

Supplement: Supplementary file 5 — Original data [file 41419_2024_6424_MOESM5_ESM.pdf]
